# Prefrontal cortical activation associated with prospective memory while walking around a real-world street environment

**DOI:** 10.1016/j.neuroimage.2022.119392

**Published:** 2022-09

**Authors:** Paul W. Burgess, James Crum, Paola Pinti, Clarisse Aichelburg, Dominic Oliver, Frida Lind, Sarah Power, Elizabeth Swingler, Uzair Hakim, Arcangelo Merla, Sam Gilbert, Ilias Tachtsidis, Antonia Hamilton

**Affiliations:** a Institute of Cognitive Neuroscience, University College London, UK; b Department of Medical Physics and Biomedical Engineering, University College London, UK; c Infrared Imaging Lab, Institute for Advanced Biomedical Technology (ITAB), Department of Neuroscience, Imaging and Clinical Sciences, University of Chieti-Pescara, Italy

**Keywords:** Functional near-infrared spectroscopy, fNIRS, Frontal lobe, Prospective memory, Social neuroscience, Ecological

## Abstract

Rostral PFC (area 10) activation is common during prospective memory (PM) tasks. But it is not clear what mental processes these activations index. Three candidate explanations from cognitive neuroscience theory are: (i) monitoring of the environment; (ii) spontaneous intention retrieval; (iii) a combination of the two. These explanations make different predictions about the temporal and spatial patterns of activation that would be seen in rostral PFC in naturalistic settings. Accordingly, we plotted functional events in PFC using portable fNIRS while people were carrying out a PM task outside the lab and responding to cues when they were encountered, to decide between these explanations. Nineteen people were asked to walk around a street in London, U.K. and perform various tasks while also remembering to respond to prospective memory (PM) cues when they detected them. The prospective memory cues could be either social (involving greeting a person) or non-social (interacting with a parking meter) in nature. There were also a number of contrast conditions which allowed us to determine activation specifically related to the prospective memory components of the tasks. We found that maintaining both social and non-social intentions was associated with widespread activation within medial and right hemisphere rostral prefrontal cortex (BA 10), in agreement with numerous previous lab-based fMRI studies of prospective memory. In addition, increased activation was found within lateral prefrontal cortex (BA 45 and 46) when people were maintaining a social intention compared to a non-social one. The data were then subjected to a GLM-based method for automatic identification of functional events (AIDE), and the position of the participants at the time of the activation events were located on a map of the physical space. The results showed that the spatial and temporal distribution of these events was not random, but aggregated around areas in which the participants appeared to retrieve their future intentions (i.e., where they saw intentional cues), as well as where they executed them. Functional events were detected most frequently in BA 10 during the PM conditions compared to other regions and tasks. Mobile fNIRS can be used to measure higher cognitive functions of the prefrontal cortex in “real world” situations outside the laboratory in freely ambulant individuals. The addition of a “brain-first” approach to the data permits the experimenter to determine not only when haemodynamic changes occur, but also where the participant was when it happened. This can be extremely valuable when trying to link brain and cognition.

## Highlights

• Neuroimaging of people while walking around London, England.
• Greater activation in prefrontal cortex when maintaining social future intentions.
• A brain-first analysis approach provided additional explanatory power.
• Functional neural events in prefrontal cortex occurred at different locations on the street. .

## 1 Introduction

Over the last 150 years, one of the major methodological challenges to understanding the functions of the prefrontal cortex has been that this brain region supports mental processes that are involved with behaviour that is voluntarily *self-initiated* (Burgess et al., 2009) and *stimulus independent* (Burgess et al., 2007). Self-initiated means that the person has decided for themselves when to act, speak, or think about something, and stimulus-independent means that the decision or thought has not been prompted by a stimulus currently available in the environment. A huge amount of our mental activity in everyday life consists of self-initiated, stimulus-independent mental processing. For instance, if we decided on the way home from work to stop and buy some milk, and this decision was unprompted by seeing or hearing a cue, then that decision is an example of stimulus-independent thought, and the act of stopping the car to shop is self-initiated. This type of mental behaviour has been shown in part to be supported by rostral prefrontal cortex (broadly, Brodmann area (BA) 10, also variously termed anterior prefrontal cortex or frontopolar cortex) (Burgess et al., 2011; Burgess and Wu, 2013), and the assessment of these processes has provided a methodological conundrum to neuroscientists and neurologists alike. Mesulam (1986) summed up this problem in relation to attempts to measure impairment of executive functions supported by the prefrontal cortex (PFC) in a neurological clinic setting. His contention was that the structured nature of the clinic setting, and its strict and obvious social and behavioural conventions, meant that impairments in these executive voluntary self-initiated processes were not required in that situation, so they were hard to demonstrate.

Mesulam's observations apply equally to the observation of these processes by neuroimaging: such processes, and their cortical substrates, are also hard to measure well using conventional neuroimaging techniques such as fMRI. This is because the participant is usually physically constrained to a degree (e.g., laying down in a scanner), the instructions and demands of the experimental situation are typically made very plain to the participant, and the event-related designs typical of the field tend to rely upon multiple repetitions of a stimulus which minimises both the stimulus-independent nature of the mental processing and the self-initiation of the responses, action or thoughts that are the object of study. For these reasons, it is hard to recreate a situation in e.g., an fMRI suite, which taps the processes involved with stimulus-independent, self-initiated thought. By contrast, there are many naturalistic situations in everyday life that do, since they are open-ended or “ill-structured”, and where the participant decides for themselves what they do and when (Burgess and Stuss, 2018; Shallice &Cooper, 2011). (A note on terms: “real-world” is used here to refer to experimentation outside the lab, in everyday situations, whereas “naturalistic” refers to experimentation that attempts to mimic situations encountered in everyday life but might be conducted in a lab. So, the experiment here is both naturalistic and conducted in a real-word environment.)

A potential solution to reduce this measurement problem therefore is given by the use of neuroimaging technologies where the temporal resolution is high, allowing near-continuous neural monitoring, and which impose few physical restrictions upon the participant; in particular, mobile (wearable) neuroimaging. Such technologies offer the possibility of studying the brain in more open-ended naturalistic circumstances which mimic everyday life and may be relatively free of the implicit or explicit constraints of the lab that minimise involvement of the mental processes under study. Here, we use fNIRS (functional near-infrared spectroscopy) to capture brain activity in an outdoors naturalistic environment. Secondarily, once freed from the constraints of being in a lab, one may also then study the relations between the spatial location of the individual and the patterns of activation in the brain—analogous to single-cell recordings from rats in a maze—that has been so successful for elucidating the functions of other brain areas such as the hippocampus (e.g., Nielson et al., 2015; O'Keefe, 1976).

The problem then however becomes how to identify the neural events: the timing of stimuli and activities in the real world is not determined by the experimenter and cannot be guaranteed in advance. Pinti et al. (2015) presented a proof-of-principle study that introduced a new analysis for fNIRS data that can potentially be used in such situations. Namely, the Automatic IDentification of functional Events algorithm (AIDE) is based on the general linear model (GLM) least square fit analysis and identifies in the time series the principal time points of neural activation within each channel (i.e., neuronal location). Accordingly, in this study, we combine mobile wearable fNIRS with both conventional experimental condition (boxcar) contrasts and also the application of the AIDE method to the data to identify neural “events”. These two methods work together well in terms of being able to make inferences from the data: The condition contrasts enable us to isolate the brain regions associated with the forms of mental processing we wish to study, and then the AIDE procedure allows us to identify where in the environment these activations occurred. The type of cognition we investigate here is prospective memory. This form has been chosen because it involves a considerable amount of self-initiated, stimulus-independent cognition. Prospective memory is the field of enquiry that concerns how, why, and when people are able to carry out intended actions after a delay period during which they have been engaged in a different activity. In other words, it concerns *delayed intentions*. Without a prospective memory ability, we would only be able to achieve our goals and objectives at the very moment we are thinking about them, and would find it very hard to put off doing something until a more appropriate time.

Our daily lives are typically peppered with numerous examples of our use of prospective memory abilities. For instance, remembering to buy some milk on the way home, or to take some medication, or to take something out of the oven when cooking. These particular examples of delayed intentions do not closely involve another person; however, there are many that do. For instance, remembering to telephone a colleague after a meeting, or to pass on a message to another person the next time you see them, or to buy a present for someone are all examples of “prosocial” prospective memory. There is good evidence that these prosocial intentions have a special status for humans. We rate them as particularly important, and are more likely to remember to carry them out (Brandimonte et al., 2010; D'Angelo et al., 2012; Kvavilashvili, 1987; Penningroth et al., 2011). So, a secondary aim of this study is to discover if there is a neural signature to this “social intention superiority effect” in a naturalistic and ecological setting.

But what relations between *spatial location* and brain activations might be expected, and how might these help identifying brain-cognition relations? Well, there are two parts to this question: the location in the brain where the activation occurs, and the physical location of the person in the environment when it happens, which of course in a moving subject would be expected to bear a relationship with the task being performed and the significance of the environment for it. Addressing these in turn, we have known for around 20 years now that activation of rostral prefrontal cortex (BA 10) is common during performance of prospective memory tasks (see Burgess et al., 2011 for historical review). The earliest neuroimaging studies used positron emission tomography (e.g., Okuda et al., 1998; Burgess et al., 2001, 2003) and so were necessarily limited in the degree to which they could investigate event-related activations. However, as it turned out, the rostral PFC activations were not strongly related to the appearance of prospective memory cues – they could occur even when cues were expected but not actually encountered (Burgess et al., 2001). Many neuroimaging studies since have replicated and extended these findings using e.g., fMRI and fNIRS (e.g., Dong et al., 2017, 2019; Halahalli et al., 2015; Martin et al., 2007; Oksanen et al., 2014), and the BA10-prospective memory link has also been confirmed by human lesion studies (Burgess, 2000, Shallice and Burgess, 1991, Volle et al., 2011), which shows that this brain region is not just involved in prospective memory, but is also necessary for it. Therefore, differences in activation of this region are an obvious place to start when considering a region that would be the one that might be most closely associated with a social intention superiority effect, should it be found behaviourally. Certainly, rostral PFC may play a role in certain social functions. For instance, rostral PFC has been found to be activated when people are telling lies to another person, face to face (Pinti et al., 2021).

This leaves the question of considering the temporal and spatial aspects of where these activations might occur. This is a much more complex matter, but there are some theories within prospective memory which can provide constraints for predictions. Within this field (prospective memory) there are three predominant theories that might be used in this respect. The first is the Spontaneous Retrieval Theory (e.g., McDaniel et al., 2004). According to this theory, a prospective memory intention is triggered spontaneously by exposure to the cue. If rostral PFC activations index such processing, activations in this region are most likely to occur in close temporal proximity to PM cue encounter. However, if PM intention retrieval also occurs frequently before intention enactment (i.e., before the cue is encountered) then there is little reason from this theory to suppose that the brain activations associated with it will not be idiosyncratic, i.e., will not follow a consistent pattern across participants.

A second influential theory is the Preparatory Attentional and Memory (PAM) processes theory (e.g., Smith, 2003). This holds that preparatory processes are engaged in maintaining a state of readiness to perform an intended task, involving monitoring of the environment for PM targets. If this form of processing is indexed by rostral PFC activations during PM paradigms, then—since the participants do not know when the PM cues will be encountered—the activations would be likely to be seen irrespective of the PM cues, temporally and spatially. A third highly influential information processing theory of prospective memory is called the Multiprocess Theory (McDaniel and Einstein, 2000). This combines both types of processing above, with differential involvement depending on the particular situation. This would predict clusters of activation around cues, but different activations (perhaps) in the intervals between spontaneous retrievals, as the environment is monitored (by a different process). In this way, these three theories make somewhat different predictions about the temporal and spatial nature of the brain activity associated with activating or maintaining a prospective memory intention. So, an aim of this study is to examine the patterns of activations in time and space and see which account best fits the activation patterns. To maximise the naturalistic nature of the experimental situation, the experiment was conducted outside, on a typical London street, with participants free to move and act as they would normally.

## 2 Method

### 2.1 Participants

Twenty-five participants took part in the present study, recruited via email to a UCL research participant database plus use of social media. Written consent was gained, and the protocol was approved by the UCL local research ethics committee (approval number CEHP/2014/901). Participants were chosen according to the following criteria:

1 18-39 years of age;
2 No history of psychiatric and/or neurological disorder;
3 Normal or corrected-to-normal vision;
4 Having not been a participant in brain stimulation studies within the last 48 hours;
5 The participants had to be comfortable walking around outside, in full view of the public, while wearing the fNIRS unit, and accompanied by the experimenters.

However, six of the twenty-five participants were subsequently excluded from the analysis. One participant performed the experiment so fast that we couldn't record accurate behavioural data on them. One participant was excluded due to human failure in setting up the fNIRS system to record data. One participant was excluded because they did not complete the entire testing procedure. Three participants were excluded because fNIRS data quality was poor, mostly due to poor light shielding and excessive sunlight generating noise in the recorded light intensity signal. Of the resulting nineteen participants who comprise the participant group reported here, ten were native English speakers, two had Polish as their first language, two had French, and the remaining five spoke Hebrew, Bulgarian, Italian, Lithuanian and Danish as their first language respectively. All were fluent in English. Out of all the participants, sixteen were students, two were employed in administrative positions, and one was unemployed. The mean age was 26 years (SD = 6.65; range = 18-39). Six were male.

### 2.2 Task conditions, order, and design rationale

In order to maximise the chances that our paradigm actually measures prospective memory (i.e., has good construct validity), and therefore that our results might be relatable to existing neuroimaging data taken from laboratory-based prospective memory investigations, we designed a task that not only mimics an everyday life social interaction in a freely-moving “outside the lab” environment, but also has the defining characteristics of a laboratory prospective memory task. These characteristics were first described in a neuroimaging experiment by Burgess et al. (2003), and were later refined by Gonen-Yaacovi and Burgess (2012). They are as follows:

1 There is an intention, or multiple intentions (Kliegel et al., 2000), upon which to act.
2 The intended act cannot be performed immediately after the intention has been formed.
3 The intention is to be performed in a particular circumstance, called the “retrieval context” (Ellis et al., 1999). This can be marked by an external cue, in event-based paradigms, or a particular time, or certain duration, in time-based paradigms.
4 The delay period between creating the intention and the appropriate time to act (i.e., the “retention interval”) is filled with an activity called the ongoing activity (Ellis et al., 1999).
5 Performance of the ongoing task prevents continuous, conscious rehearsal of the intention over the entire delay period. This is typically because the ongoing activity places a heavy demand on competing cognitive resources or the delay is too long.
6 The PM cue does not interfere with, or directly interrupt, performance of the ongoing task. Intention enactment is therefore self-initiated (Graf and Uttl, 2001) and, thus participants are required to recognise the PM cues or retrieval context themselves.
7 In most situations involving PM no immediate feedback is given in response to the participants’ errors or other aspect of performance.

**Fig. 1:**
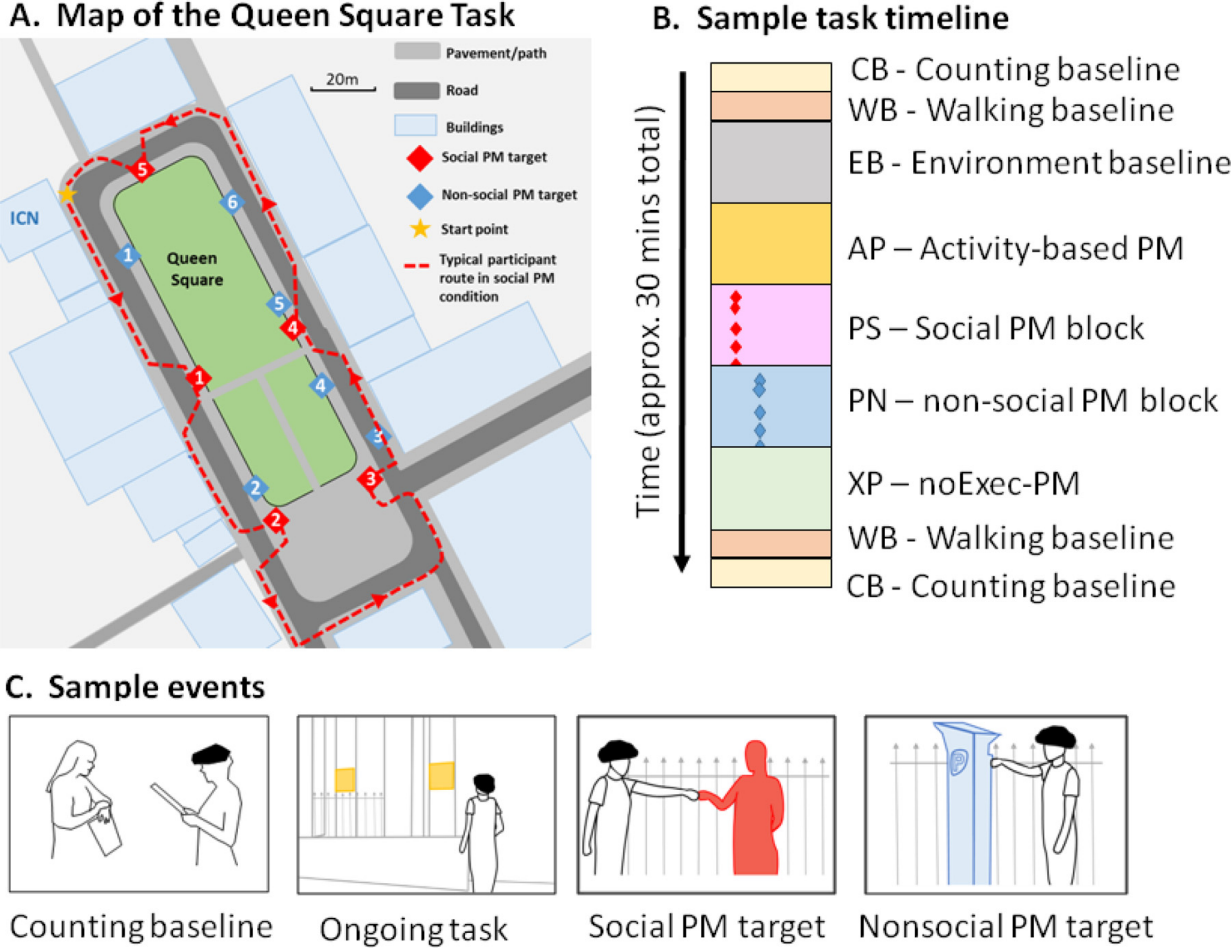
(A) Map of the Queen square task showing a typical participant trajectory. (B) A sample task timeline. PM events where the participant encounters a PM target are shown as coloured diamonds. (C) Sample events including the baseline tasks, the ongoing task (participants must search for notices on buildings highlighted in yellow), the social PM task (participants must fist-bump a confederate) and the non-social PM task (participants must fist-bump a parking meter). (For interpretation of the references to color in this figure legend, the reader is referred to the web version of this article.).

It is important to note that none of these characteristics is unique to a situation that taps prospective memory. It is the combination of them that distinguishes a PM task from e.g., a task-switching or “working memory” one. These principles determined the design of our experimental paradigm. In addition, we added a series of baseline contrast conditions in order to aid with interpretation of the resulting changes in fNIRS signals. Tachtsidis and Scholkmann (2016) point out that a fundamental aspect to consider when designing an fNIRS protocol is that haemodynamic changes must be assessed relative to a control condition or baseline, and that the experimental protocol needs to have the best possible baseline condition to subtract out “spurious” hemodynamic/oxygenation responses from the experimental task, thereby creating a high contrast between either an experimental condition and a baseline condition or between two experimental conditions. Accordingly, we utilised several baseline conditions which together aimed to sample from the information processing systems that are required to perform the task (e.g., counting, walking, remembering instructions etc.) but are not of interest to the hypotheses under consideration. Each testing session was divided into nine blocks or conditions which we refer to with 2 letter acronyms (see Fig. 1 and below). Six of these blocks (3 conditions, with two of them repeated once) were concerned with measuring haemodynamic responses related to the basic or baseline activities required by the task. These three baseline activities were (a) counting (CB), (b) walking (WB), and (c) inspecting the Queen Square environment (environmental baseline, or EB). Two of these (counting and walking) are behaviours that are required during all the task blocks, but are of no interest for the current study, so these baseline conditions permit exclusion at the analysis stage of neural responses related to them. In addition, the environmental baseline condition (EB) where the participant walked once around the Queen square testing space was used to ensure that the neural responses seen in the first experimental condition would not be substantially related to the novelty of inspecting the environment rather than the task demands per se. In other words, it afforded environmental familiarization (see Wilkinson et al., 2006 for discussion of such experimental design features): The issue of novelty is a complex matter for measurement of frontal lobe function (Burgess, 1997). For this latter reason, we also did not use a large number of PM execution events. It is proposed within the prospective memory (PM) literature that the construct validity of a PM task may decrease with increasing repetition as a function of routinisation of association between cue and behaviour (Gonen-Yaacovi and Burgess, 2012).

In addition to these baseline conditions, every participant performed four experimental task blocks. Each involved walking one circuit of Queen Square while performing one ongoing task, plus the activity-based PM demand (to stop and say “clear for crossing” before crossing a road). Two of these blocks also required participants to perform an event-based PM task in addition. This follows the convention in the field of prospective memory, where an ongoing condition involves the same type of task as the prospective memory condition, but has no delayed intention component (e.g., Burgess et al., 2001). The first task block was always an ongoing task with only the activity-based PM demand (i.e., no social or non-social event-based PM demand). We refer to this as the activity-based PM condition (AP). Following this block, participants completed two event-based (plus activity-based) PM task blocks, one with a social PM task (PS) and the other with a matched non-social task (PN). During the event-based PM conditions the participants were engaged in an ongoing task, but in addition they were given a prospective memory instruction that made an event-based delayed intention requirement (see below for description). After this, participants completed a fourth experimental block (no-Exec PM or XP) that was a repeat of the activity-based PM condition, and the participants were told *not* to respond to event-based PM targets (i.e., the confederate/parking meters, see below), even though they would be available just as in the social and non-social (PS and PN) blocks.

### 2.3 Location

Set-up for the Queen Square prospective memory task took place in a testing room at the UCL Institute of Cognitive Neuroscience, Queen Square, London, UK. Participants first gave written, informed consent to take part in the study in accordance with Research Ethics requirements. The limits of Queen Square (QS) formed the boundaries of the testing area. Participants were instructed not to walk down side streets or out of the Queen Square area, nor were they allowed to enter the gated park in the centre (see Fig. 1A). This meant that participants could return to the starting point by walking in a ‘loop’ in one direction around the square. Participants were told not to turn back on themselves, but were otherwise permitted to roam freely.

### 2.4 Experimental procedure

Before entering the testing area, participants were taught the task rules, repeating a presentation-free recall procedure until a criterion of one fully correct recall was reached. Once entering the testing area, but before testing started, this procedure was repeated. Errors and omissions were recorded. Participants were allowed to ask questions only during the instruction periods before the ongoing and PM tasks began, and were told to go “as fast as you can without making mistakes” and without leaving the experimenters behind. Details of the conditions are as follows:

#### 2.4.1 Counting baseline (CB)

Participants were given a laminated A4 sheet of paper on which were printed a sequence of the characters ‘X’ and ‘O’. These were randomly ordered with a ratio of 5:1 (X:O). Participants were asked to count the number of ‘O's on the sheet of paper as quickly as possible without making mistakes. There were two sheets, one with a total of 64 ‘O's and one with 72 ‘O's printed on it. One sheet was given at the beginning and the other at the end. The order of the sheets was counterbalanced between participants. Participants were timed with a stopwatch until they responded verbally with their answer.

#### 2.4.2 Walking baseline (WB)

Participants were asked to walk at a natural walking pace from the starting point down the street whilst counting 60 paces in their head. Once they reached 60 paces, they were asked to stop, notice how far down the street they had reached, turn around and walk another 60 paces back to the starting point. The experimenter noted how many steps were actually taken.

#### 2.4.3 Environmental baseline (EB)

From the starting point, the boundaries of the testing area were explained by leading the participant around Queen Square in an anticlockwise direction at a normal walking pace. Two features of the environment (parking meters and a confederate) were used as targets in the PM blocks (see below). Since parking meters could not be temporarily removed, to mitigate the possibility of the participant becoming more familiar with non-social targets than social targets, the social confederate (see PM social condition) silently accompanied the experimenter on this walk, and so was also present for the duration of this baseline activity. Each possible point of departure from the square was indicated as out of bounds, as was the park. Before crossing a delivery entrance in the North West corner of Queen Square, which is immediately before the starting point, participants were warned to take care when crossing the road. This was to indicate that the participant should treat this as a road crossing later during the experiment.

#### 2.4.4 Ongoing tasks

In the parlance of prospective memory theorists, ongoing tasks are the tasks that occupy the participant during the delay period between creating a delayed intention and its execution (or expected execution). In this study, participants were given four ongoing tasks to complete in Queen Square during the four experimental blocks (Activity-based PM (AP), Social prospective memory (PS), non-social prospective memory (PN) and No-execution PM (XP)). The order of these tasks was fully counterbalanced across participants. All four tasks involved counting items consistently found on the participant's right-hand side (the opposite side of the road to the PM targets) (Fig. 1C). Pilot studies indicated that the following items were roughly commensurate for difficulty. Participants were asked to walk round Queen Square and determine the number of:

a) Signs on buildings that contain the word “Queen”.
b) Days, dates and opening hours displayed on buildings.
c) Unobstructed stairways (not single steps).
d) Push-button entry systems at ground floor level.

Written instructions for each task, which included example photos from real life environments other than Queen Square, were printed on laminated A4 sheets of paper and given to the participants and the instructions were read aloud by the experimenter between each block. There were two episodes of administration of these ongoing tasks, when the participants were not required to make a PM response (i.e., no fist bumps).

#### 2.4.5 Activity-based PM (AP)

Upon returning to the starting point at the end of the Environmental Baseline, participants were given a new “road crossing” rule. Participants were instructed that each time they wanted to cross a road, they should first say “clear for crossing” clearly to the accompanying experimenter. Only when the experimenter had replied “clear” were they allowed to cross. The participant was asked to repeat this rule to ensure that they had understood it correctly. This requirement acted as an activity-based PM task (it was also required for safety purposes) and was also present during the event-based conditions (social and non-social prospective memory). Finally, it was explained that the experimenter would walk slightly behind the participant at all times during the experiment to “not get in [their] way.” This was to minimise potential experimenter effects. Participants were also told that they would be asked to slow down if they were walking too fast. The ongoing tasks as described above were administered in the activity-based PM block *before* any mention of the event-based social or non-social prospective memory instructions were given. So, in this condition, the participants were naïve to the event-based component of the experiment, and were performing the ongoing tasks, plus an activity-based PM component (i.e., the road-crossing procedure).

#### 2.4.6 Event-based prospective memory: social (PS)

In both the social and non-social event-based prospective memory conditions, participants were engaged in an ongoing task (see above), and also had the activity-based instruction (road crossing, as in condition AP above) but in addition were given a PM instruction, which was to respond to a particular target if it was seen. The social PM target was one of the experimenters (the “confederate”) who moved into various positions around Queen Square as the task progressed, in such a way that they would be out of sight while they moved into position. The positions for the social confederate to stand were on the participant's left-hand side and typically required the participant to cross the road to reach the location (Fig. 1A). Participants would therefore not necessarily see the PM targets without remembering to look for them. This was designed to mimic a ‘real-life’ situation, in which a self-initiated search process is typically required for successful PM retrieval.

Participants were given the event-based PM instruction after having been told which ongoing task they would perform during that block. The experimenter said “This time, there is something else you must do as well. If when you are walking around, you come within 50 metres of this experimenter [indicating the confederate] we would like you to go over to her and clearly but gently bump her with your fist like this.” A ‘fist-bump’ action (commonly used as a greeting in some social circles) was then demonstrated with the social confederate (see Fig. 1C). Participants were told that if they came across the confederate more than once, they were to respond whenever they came within 50 metres of her and not just once. Different experimenters took on the role of the social confederate for each participant. During this condition, the participant would have the opportunity to “fist-bump” the confederate 5 times (each time in different locations; see Fig. 1).

#### 2.4.7 Event-based prospective memory: non-social (PN)

The non-social prospective memory condition was identical to the social one except that parking meters were used as the PM target rather than the confederate. Participants were engaged in one of the ongoing tasks but were told also that if they came within 50 metres of a parking meter, they should go over to it and “fist-bump” it (see Fig. 1C). Thus, the intention (to go to the target and fist-bump it) and the actual PM action (the fist-bump) were identical between the social and non-social conditions. As with the social PM locations, parking meters were found mainly on the participant's left-hand side and the participant had to actively search to see them. There were a similar number of opportunities for the participants to fist-bump parking meters as to fist-bump the confederate (see Fig. 1; the actual number of parking meters differed slightly for some participants during the data collection period because of building works on the street that were beyond our control).

#### 2.4.8 No-execution PM condition (XP)

This condition was identical to the event-based PM block except that this time participants were explicitly told that they should not respond to PM cues. The PM cues they had previously responded to were present, however: The parking meters were always present in the testing environment (since they were real parking meters fixed to the ground) and the social confederate assumed the same five positions in this condition as in the social PM condition. However, the activity-based PM demands of this condition remained, and so was equivalent to the activity and event-based PM conditions, with the chief difference being that these PM cues were not to be acted on (i.e., no intention execution).

mean number of events and the mean durations of the conditions are given in Table 1 and The specification of the assumed information-processing systems involved in each of these conditions given in Table 2 (see below for further details). We presumed 9 sets of information-processing systems which may not be neurally identical in representation. These were those involved in: walking; numerical processing (e.g., counting) and maintaining and updating a number; visual search for targets/stimuli in the environment; specialised social PM intention maintenance; specialised non-social PM maintenance; generalised processes involved in maintaining any event-based PM intention (represented by PS+PN); intention execution (e.g., fist bump); activity-based PM execution, represented by road crossings; verbal learning (activity during learning the tasks rules); rule-breaks (failures to say “clear for crossing” when crossing the road). The logic of the contrasts used in the fNIRS analysis relates to this specification.

**Table 1:** Design matrix conditions with event counts and durations. The two letter acronyms match the condition labels in Fig. 1.

Columns of the design matrix	mean events per participant	mean duration
Counting baseline (CB)	2	30.5 sec
Walking baseline (WB)	2	69.8 sec
PM events (PE)	30	9.8 sec
Null events (NE)	20	53.2 sec
Environment Baseline (EB)	1	335 sec
Activity-based PM (AP)	1	417 sec
No-execution PM (XP)	1	403 sec
Social PM block (PS)	1	569 sec
Non-social PM block (PN)	1	552 sec
Rule recall (RR)	3	26.3 sec
Rule break (RB)	5.5	4.9 sec

**Table 2:** Information processing level specification of the condition contrasts.

Measure	Walk	Number process and remember	Search for external targets	Event-based Social intention	Event-based Nonsocial intention	Intention execution	Activity-based intentions (road crossings)	Verbal learning	Rule break
Counting baseline (CB)		✓							
Walking baseline (WB)	✓	**✓**					✓		
PM events (PE)	✓	✓	✓	✓	✓	✓	✓		
Null events (NE)	✓	✓	✓	✓	✓		✓		
Environment Baseline (EB)	✓								
Activity-based PM (AP)	✓	✓	✓				✓		
No-execution PM (XP)	✓	✓	✓				✓		
Social PM block (PS)	✓	✓	✓	✓			✓		
Nonsocial PM block (PN)	✓	✓	✓		✓		✓		
Rule recall (RR)								✓	
Rule break (RB)									✓

#### 2.4.9 Condition ordering

The condition orders for the four baseline conditions (walking, counting, environmental baseline and rule learning and rule recollection) were fixed for every participant, with all four baselines at the start of the task and the walking and counting conditions additionally repeated at the end. The four main task blocks each with one circuit of Queen Square were combined with four matched ongoing tasks as detailed above. The first block of the ongoing task is always analysed as the activity-based PM (AP). The next two blocks were always the PM blocks, and the order of the conditions (PS, PN) was counterbalanced across participants, so that half received the social condition first, and half received the non-social condition first. The last ongoing task block was always the ongoing-contaminated block (No-execution PM block, XP). The combinations of ongoing tasks and PM conditions order were counterbalanced across participants according to the Latin Square method.

### 2.5 Behaviour and fNIRS data acquisition

The instrumentation and general method of data collection have previously been described in detail in Pinti et al. (2015), where we show a video recording of the various tasks in one participant. For scoring and marking up of behavioural events, the experiment was recorded using three digital cameras and a voice recorder. A Sony voice recorder was placed in the participant's pocket in order to capture verbal responses. General direction of gaze was captured by a lightweight Mobius HD 1080p 30fps action head camera attached to the top of the fNIRS cap. Close-range footage of participant actions was filmed on a 1080p 30fps GoPro Hero camera affixed to the experimenter's chest. An additional experimenter also filmed the participant and experimenter using a handheld Sony HDR camcorder with a 29.8mm wide-angle lens from a distance of approximately two metres behind them at all times.

**Fig. 2:**
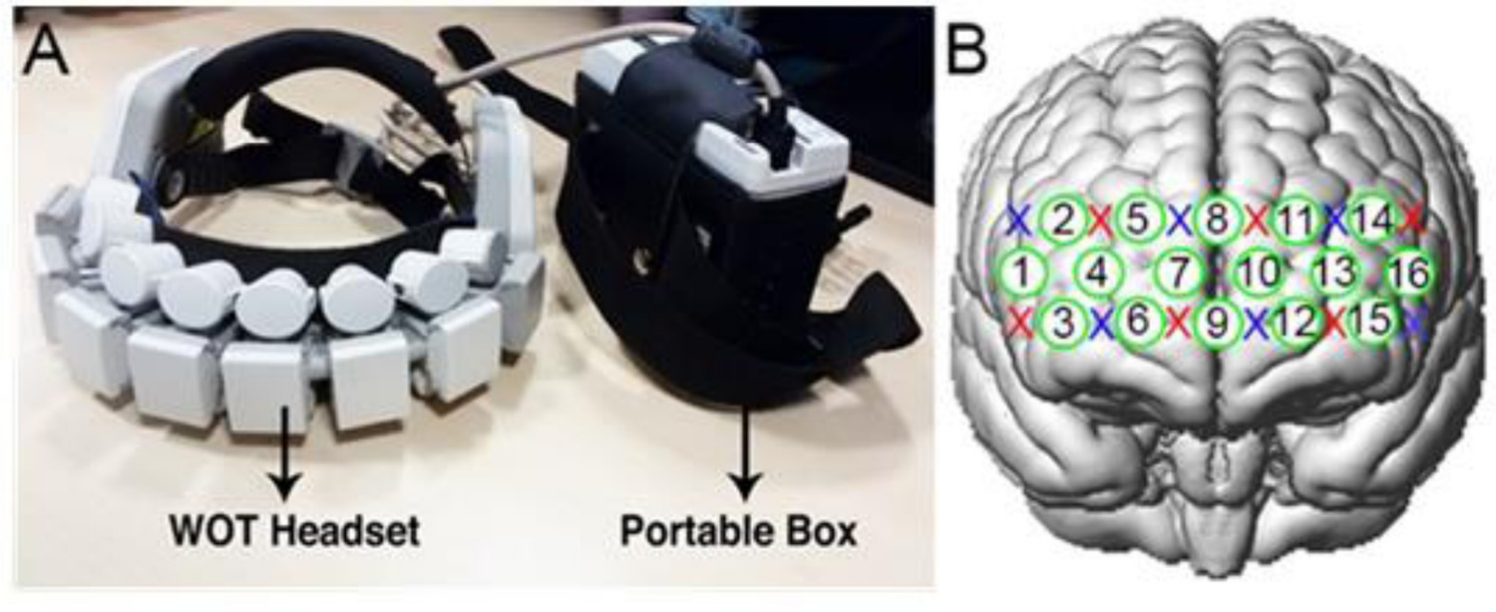
The Hitachi WOT fNIRS system and channels. Panel A shows the WOT system. Panel B shows the channel positions, where green circles represent the 16 measurement channels, and the red and blue crosses indicate the sources and detectors, respectively. (For interpretation of the references to color in this figure legend, the reader is referred to the web version of this article.).

We used the wireless Wearable Optical Topography System (WOT) fNIRS system from Hitachi High-technologies Corporation. The system is comprised of a headset and a portable box, which the participant wore at their waist (see Fig. 2, panel A). The headset consists of six light sources and six detectors arranged in an alternating geometry, creating sixteen measurement channels (Figure 2, panel B), which were placed over the prefrontal cortex. The light sources were vertical-cavity surface emitting laser diodes (VCSEL), emitting near-infrared light at 705nm and 830nm. The detectors were silicon photodiodes (Si-PD). The sampling frequency of the system is 5 Hz. The general equipment setup procedure is outlined in Pinti et al. (2015).

**Fig. 3:**
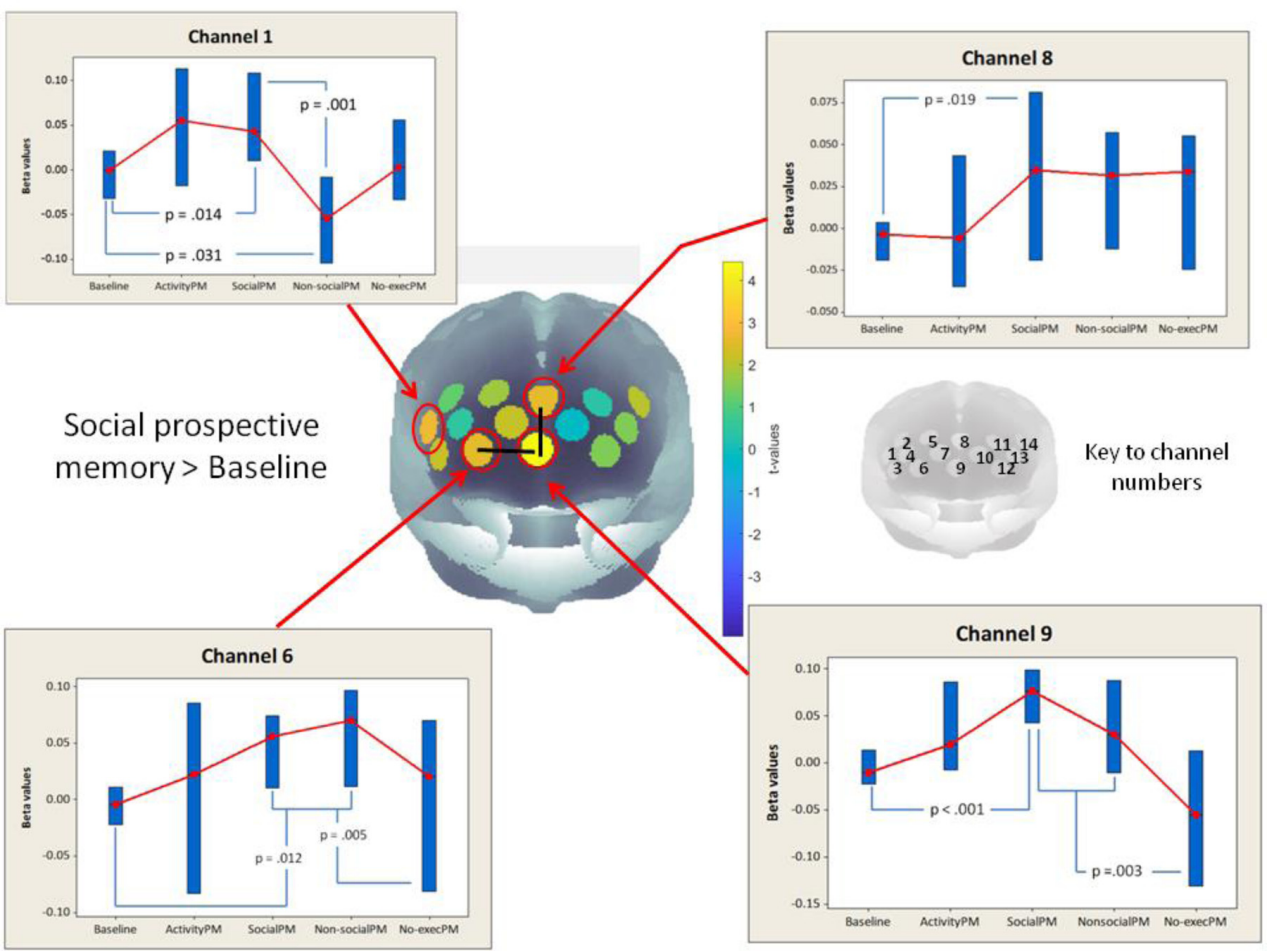
Brain activity during the social prospective memory condition compared to the baseline conditions. The correlation-based signal improvement (CBSI) preprocessing step (Cui et al., 2010) was used to combine the preprocessed HbO_2_ and HHb signals into an activation signal represented by the beta values (Scholkmann et al., 2014). Since brain activity corresponds to an increase in the activation signal, the signal was fitted with a positive HRF similar to HbO_2_ and BOLD. Therefore, functional brain activity is represented by positive beta-values given here. The baseline activity is represented by the mean of the four individual baseline conditions: counting (CB), walking (WB), environmental (EB), and recall (RR). The two channels that were identified a priori were channels 6 and 9 as indicated. These channels were also significant after correction for multiple comparisons at the array-level of analysis (indicated by a black line joining the channels). The additional channels indicated as significant were channel 8 (also significant at an array-corrected level) and channel 1 (p<.05 channel-wise). Individual panels show the patterns of activation for the channels showing significant differences across channels at a p<.05 uncorrected threshold. The bars are 95% confidence intervals for the condition medians, which are marked with a red symbol. Each panel shows the conditions in the order: Baseline, Activity-based prospective memory (ActivityPM or AP), Social prospective memory (SocialPM or PS), Non-social prospective memory (Non-socialPM or PN) and finally the conditions where all the PM targets were available, but participants were instructed not to respond to them (No-execPM or XP; see Table 2). (For interpretation of the references to color in this figure legend, the reader is referred to the web version of this article.).

In order to place the headset in a reliable way across all participants, we used the 10-20 electrode placement system and placed channel 9 onto the Fpz point and channel 8 and 9 aligned to the Fpz-Fz line. A 3D magnetic digitizer (Fastrak, Polhemus) was then employed to digitize the locations of the optodes (i.e., sources and detectors) and the 16 channels and of six landmarks (Nasion, Inion, right and left pre-auricular points, Fz, Cz). The digitized coordinates were converted into the MNI space and co-registered onto a standard brain template using the NIRS SPM software package (Ye et al., 2009). The median MNI coordinates of the 16 channels computed across all the participants and the corresponding anatomical location according to the Brodmann Areas atlas were then determined (see Zhang et al., 2017), and are given in the Supplementary Material.

### 2.6 Data analysis

Concentration changes of oxy- and deoxy-Hb were computed by the WOT processing unit using the modified Beer-Lambert law and calculated with respect to a zero-baseline period at the beginning of the experiment. Measurements are thus not corrected for the optical pathlength and are presented in (mmol * mm/l). Raw HbO_2_ and HHb data were down-sampled to 1 Hz and motion artefacts were identified and corrected using the wavelet-based approach (Molavi and Dumont, 2012) implemented in the Homer2 software package (Huppert et al., 2009). Physiological noise (e.g., heart rate, breathing rate) and slow trends in the signals were removed using a Butterworth band-pass filter between 0.008 and 0.2 Hz. The correlation-based signal improvement (CBSI) preprocessing step (Cui et al., 2010) was then used to combine the preprocessed HbO_2_ and HHb signals into the so-called ‘activation signal’ (Scholkmann et al., 2014). The CBSI method was applied for two reasons: (i) it allows us to infer functional brain activity on only one signal that includes information on both HbO_2_ and HHb; (ii) it increases the contrast-to-noise ratios of fNIRS signals reducing the impact of non-neuronal systemic confounding factors (e.g., heart rate, breathing rate, Mayer waves) that we expect to be more pronounced in HbO_2_ in freely moving people (Tachtsidis and Scholkmann, 2016). The activation signal for each of the 16 channels over the experimental conditions was then fit to an individual design matrix for each participant, using custom Matlab scripts calling the estimation functions from NIRS-SPM (Ye et al., 2009). See Tak and Ye (2014) for further review of statistical analysis of fNIRS data.

To create the design matrix, we completed the following steps. First, the three video streams for each participant were synchronised in ELAN (Brugman and Russel, 2004) and coded to identify the timings of key events and block transitions within each run. Event timings generated from the videos were synchronised with the fNIRS timeline. We then set up a design matrix to model the seven conditions illustrated in Fig. 1B (3 baselines + 4 task blocks). In addition, we modelled time points where participants were engaged with a PM target (e.g., saying ‘clear for crossing’, crossing the road, moving directly toward the target, fist-bumping the target) as PM-events with a minimum duration of 4 seconds. We modelled time-periods during the PM blocks where participants were not engaged in any PM target as null events with variable duration (minimum 4 seconds) as Baseline-events. In addition, we modelled the time-points where participants were repeating the task rules to the experimenter with the duration of that event, labelling them ‘rule recall’. Any time-points where participants broke a task rule (e.g., crossing the road without saying ‘clear for crossing’ were labelled ‘rule break’ events and modelled with their duration. Thus, each design contained 11 columns – the 7 task conditions plus PM events (PE), null events (NE), rule recall events (RR) and rule break events (RB). Every block of event was modelled as a boxcar of the appropriate duration convolved with a standard haemodynamic response function.

The rationale for this type of design matrix is as follows: Block-level contrasts between conditions aim to indicate the mental processing that occurs throughout the conditions, often in a stimulus-independent way (Burgess et al., 2007). As outlined above, examples of such mental activity are often seen during prospective memory tasks and are thought to correspond to maintenance of the intention, or an increased state of attending in anticipation of encountering a PM cue (see Burgess et al., 2001). The logic of the contrasts between conditions is similar to that used before in studies of prospective memory (e.g., Burgess et al., 2001, 2003). It is hypothesised that the prospective memory conditions (including performance of the ongoing tasks) required (a) walking, (b) counting objects and remembering the number detected, and (c) looking at and responding to the natural environment of Queen Square (e.g., people, cars, buildings, etc.,) in addition to the intention-related component. Together, the four baseline conditions aimed to measure the PFC activity relating to these background cognitive demands. So, block-level contrasts between the baseline conditions when considered together aimed to reveal haemodynamic patterns accompanying the prospective memory component of the tasks, especially that related to maintaining an intention, since it is assumed that the proportion of time in the PM conditions spent maintaining intentions is larger than the period of time spent executing them. The information-processing level specification of these block contrasts is given in Table 2.

In addition, we aimed to model activity that is specifically linked to detecting a prospective memory cue (i.e., either the parking meter or the social confederate) and executing an intention rather than maintaining it. Thus, we created the event-regressors for PM events and null events. The PM event regressors represented the period of time from 5 seconds before the participant started the execution, i.e., either the activity-based PM measure (saying “clear for crossing”) or heading towards the PM target (either the confederate or the parking meter), until after the PM intention was executed (e.g., the “fist-bump”). There were 577 of these events across the whole participant sample, with a mean duration of 9.8s (SD 8.8s). They were contrasted with a series of 380 null event blocks drawn from the uncontaminated OG, social PM, non-social PM, and contaminated OG blocks. In other words, the null events were periods of time greater than 10 seconds long where participants are walking around the testing environment and involved with the experimental conditions, but not in recognising or interacting with a PM target.

### 2.7 A priori predictions and hypotheses

To determine whether the fNIRS results from this experiment bear a strong relation to those from different methods (e.g., fMRI, PET) in very different (lab-based) environments and using very different tasks, we outlined a set of a priori hypotheses. The “typical pattern” of activations as determined by fMRI and PET during PM lab-based event-based experiments (see Burgess et al., 2011; Burgess and Wu, 2013, for reviews) is that when a person is maintaining an intention (vs. when they are doing the ongoing task only) there tend to be changes in activation in both lateral and medial rostral PFC (area 10; although the lateral and medial regions may show opposing patterns of activation). Examples can be found in e.g., Benoit et al., 2012; Burgess et al., 2001, 2003; den Ouden et al., 2005; Gilbert et al., 2009; Hashimoto et al., 2011; Okuda et al., 1998, 2007; Simons et al., 2006. We examined the median location for the activation increases in lateral and medial rostral PFC from 11 clusters from five studies (two PET, 3 fMRI) from our lab where a similar comparison has been made as in this experiment (Benoit et al., 2012; Burgess et al., 2001, 2003; Gilbert et al., 2009; Simons et al., 2006). The result was that for lateral rostral PFC the median focus was located at +/-39, 56, 4 (x, y, z MNI co-ordinates; activations tend to be bilateral). The equivalent focus for the medial rostral PFC activation changes during PM conditions was centred upon x=0, y=60, z=2 (based also upon 11 activation foci from the above named studies).

The closest relation spatially between a channel available in the Hitachi WOT system and the medial MNI template-referenced finding from previous fMRI and PET studies is Channel 9 (see Fig. 2 for channel configuration): the approximate MNI equivalent position for the WOT channel 9 is 1, 72, -2, which is close to this medial region (0, 60, 2) identified from our previous studies above. The closest equivalent for the lateral region identified from previous studies (+/-39, 56, 4) is less obvious, since whilst Channels 13 (approximate median MNI position 39, 61, 13 across all participants) and 4 (39, 61, 13) are quite close in proximity, they likely lie within a different functional and cytoarchitectural subregion, outside polar rostral PFC. Accordingly, we chose the closest channels within the same cytoarchitectonic subregion of rostral PFC as that identified in our previous studies, corresponding to area fp1 of Bludau et al. (2014). They were channels 6 and 12, the median position of which in our participants were 29, 69, -2 and -27, 68, -3 respectively, approximately 18 mm away from the median lateral co-ordinates of our previous fMRI findings.

Accordingly, to determine if our fNIRS experiment is capable of provoking, and detecting, changes in the typical regions associated with prospective memory, we examined activations in these channels as a priori selections, and also examined the specificity of them (since if all channels showed the same patterns then clearly this is less remarkable). We then proceeded to interrogate all the channels to see whether there was any brain region which was differentially active in either the social or non-social conditions.

### 2.8 Contrasts and statistics

When the design matrix for each participant had been built, it was estimated to obtain beta parameter estimates using NIRS-SPM (Ye et al., 2009). The parameter estimates were compared using paired t-tests in a similar manner to SPM. First, we considered the levels of activation in the channels chosen *a priori* to be a validation of the procedure. For these channels, a threshold of p<0.05 uncorrected on each individual channel was used, and also note where our results meet the array-level corrected threshold below. We calculated several contrasts, comparing our experimental conditions (AP,PS,PN and XP) to a baseline contrast composed of the walking baseline, counting baseline, environment baseline and rule recall events: Baseline = average (WB,CB,EB,RR).

Second, we calculated four contrasts at the whole-array level. For each of these contrasts, we consider a result to be statistically significant if two adjacent fNIRS channels show effects in the same direction with p<0.07 in both channels. Monte Carlo simulations with our specific array configuration show that this channel-level threshold gives a whole-array false positive rate of p<0.05 (Southgate et al., 2014). Thus, this gives an appropriate correction for multiple comparisons at the array-level of analysis. The five contrasts we evaluated were: Social-PM > Baseline; Nonsocial-PM > Baseline; Social-PM > Nonsocial PM; Activity-based PM > Baseline; and PM events (encountering a target or crossing a road) > null events (time period during the task without either happening), that is, PE > NE.

### 2.9 AIDE analysis

As discussed above, the AIDE algorithm (Pinti et al., 2017) is a method that was specifically developed for naturalistic applications of fNIRS. It is a “brain-first” approach, which means that the onsets and durations of significant functional events are first detected from observed signals in the brain and then this is related to the individual's situation in the environment, rather starting from events specified by a lab-based stimulus design and then relating that to what is happening in the brain at that point. More specifically, AIDE uses the GLM-based least squares fit analysis to estimate functional events from all possible temporal combinations of event onsets and durations and, then, an iterative procedure assesses the resultant *β*- and *t*-values corresponding to these GLMs, with the retained t-values forming a time-series from which peak-values are be extracted. Statistically significant functional events represent the best fit between the observed fNIRS signal and AIDE activation model. Results are corrected using the False Discovery Rate (Poldrack et al., 2011). The validity of AIDE-derived activation models depends in large part on whether or not the detected functional events that yield them correspond to *task-relevant* information processing pathways in the brain. Therefore, AIDE was applied to the cleaned CBSI signals of the channels covering areas of rostral PFC (BA 10 [channels 6, 8, & 9]), and right inferior and middle frontal gyri (BA 45/46 [channels 1, 3], respectively) to investigate the extent to which recovered functional events were indicative of the patterns of activation that would be expected from the resource requirements of subsystems supporting stimulus-oriented and -independent attending in prospective memory situations. There were three measures by which this was assessed: event location, event frequency, and event rate.

*Event location* refers to where in the environment functional events occurred. To obtain these data, the functional event timings were linked back to the video footage collected during the experimental task. These event locations can then be interpreted in relation to the task landmarks (roads/PM targets etc.). The onsets of these events should be detected where participants are either engaged in task-related actions in the environment (e.g., interacting with another person) or in key information processing steps relating to the task (e.g., spontaneously retrieving the intention to interact with another person upon sight). The distribution of these event onsets over time and conditions should sufficiently indicate this engagement (e.g., group-level aggregations of functional events in relevant spatial locations).

*Event frequency* refers to the degree to which functional events are extracted from different brain subregions throughout the time-series, and whether the frequency of functional events between and within regions exhibit greater or lesser frequencies is the *event specificity*. Predictions about these parameters should depend on the hypotheses of individual paradigms. For example, for the present one, this measure of incidence should theoretically be greatest in the subregion of interest with respect to PM tasks (i.e., BA10).

*Event rate* refers to the temporal change in functional events throughout the course of a given task. If neural events index a particular class or form of cognition, and in theoretical terms it is expected that these cognitions will be occurring only at certain times, then the incidence of them might be expected to aggregate at certain times. One means by which such task-related variation in event rate can be demonstrated is to show that some random process does not equally account for it, such as a Poisson random variable (see Gabbiani and Cox, 2010). That is, at least some events should not be independent from each other. To test whether event rates were not random, synthetic null distributions of Poisson arrays were produced using the standard Matlab function ‘poissrnd’ from the Statistics and Machine-Learning Toolbox. This approach used the real data vectors of event onsets and the means of their rates as input to simulate random event rates.

## 3 Results

### 3.1 Behavioural performance

In the 4 ongoing tasks (e.g., detect a sign with the word ‘Queen’ or a push-button doorbell), participants on average detected 70.8% of the items that they should have detected (SD 7.4%, range 53.5%-83.5%). Performance was not significantly affected by task experimental order or PM block (both p > 0.1, ANOVA). Ongoing task performance in social PM conditions was a little weaker than in the non-social PM condition (social: mean 65.8% correct, SD 15.5%; non-social: mean 71.7% correct, SD 16.7%), but this difference was not significant at p<.05. In the two event-based PM tasks, participants were instructed to notice and fist-bump a confederate or a parking meter. There was no significant difference between the PM social and PM non-social conditions in terms of the number of PM targets that were responded to (PM non-social performance (mean, SD) = 86.8%, 16.7%; PM social performance = 89.5%, 20.4%; paired t-test).

### 3.2 fNIRS results

Channels 15 and 16 (situated in left lateral PFC) had to be excluded from the analyses as the results were corrupted by poor light shielding in more than the 50% of the participants. (The optical detector for these two channels was located at the bottom left corner of the WOT headset (Figure 2B), near the left temple). To create a baseline to which the PM conditions could be compared, we averaged across the baseline conditions: walking baseline, counting baseline, environmental baseline and the rule recall (see above). This is because all the PM conditions involve, walking, counting, navigating round the environment, and recalling task instructions. So, this measure will be referred to as the Baseline in this Results section.

#### 3.2.1 A priori hypotheses

We first considered the most medial of the channels chosen a priori, (channel 9; medial frontopolar BA 10, approximate MNI co-ordinates 1, 72, -2. This region showed a significant increase in activity from baseline in the social prospective memory condition (t = -4.45, p = <.001), and a significant decrease in activity from both event-related PM conditions to the no-execution PM condition (t = 3.59, p = 0.002; see Fig. 3). None of the other contrasts between conditions were significant.

For the two lateral BA 10 regions identified a priori, the pattern of activations was very different according to the lateralisation. For the right hemisphere channel (channel 6, right BA10/11/47), there was significantly higher activation during both event-based prospective memory blocks (for social, t = 2.65, p = 0.016; non-social t = 2.34, p = 0.031), with no other comparisons significant at p<.05 (Fig. 3). For the equivalent channel in the left hemisphere (channel 12), no conditions showed activations that approached being significantly different from the baseline (the largest difference between any two conditions was between PM social and Activity PM, t = -1.73, p = 0.100.) Thus, the lateral BA 10 PFC increases in the event-based prospective memory conditions seemed to lie within the right hemisphere only.

Interestingly, both the right lateral and medial BA10 channels (6 and 9) showed a significant decrease in activation in the no-execution PM condition, where the only difference between that condition and the event-based prospective memory ones was that participants were told not to respond to the PM targets (the confederate or parking meters). Overall, then, of the three channels identified a priori on the basis of fMRI and PET studies as likely candidates for significant activation changes during event-based prospective memory conditions, two of these regions did indeed show significant changes over baseline levels, showing how specific the activations are to the intention to carry out an intention.

#### 3.2.2 Whole-array contrasts

**Fig. 4:**
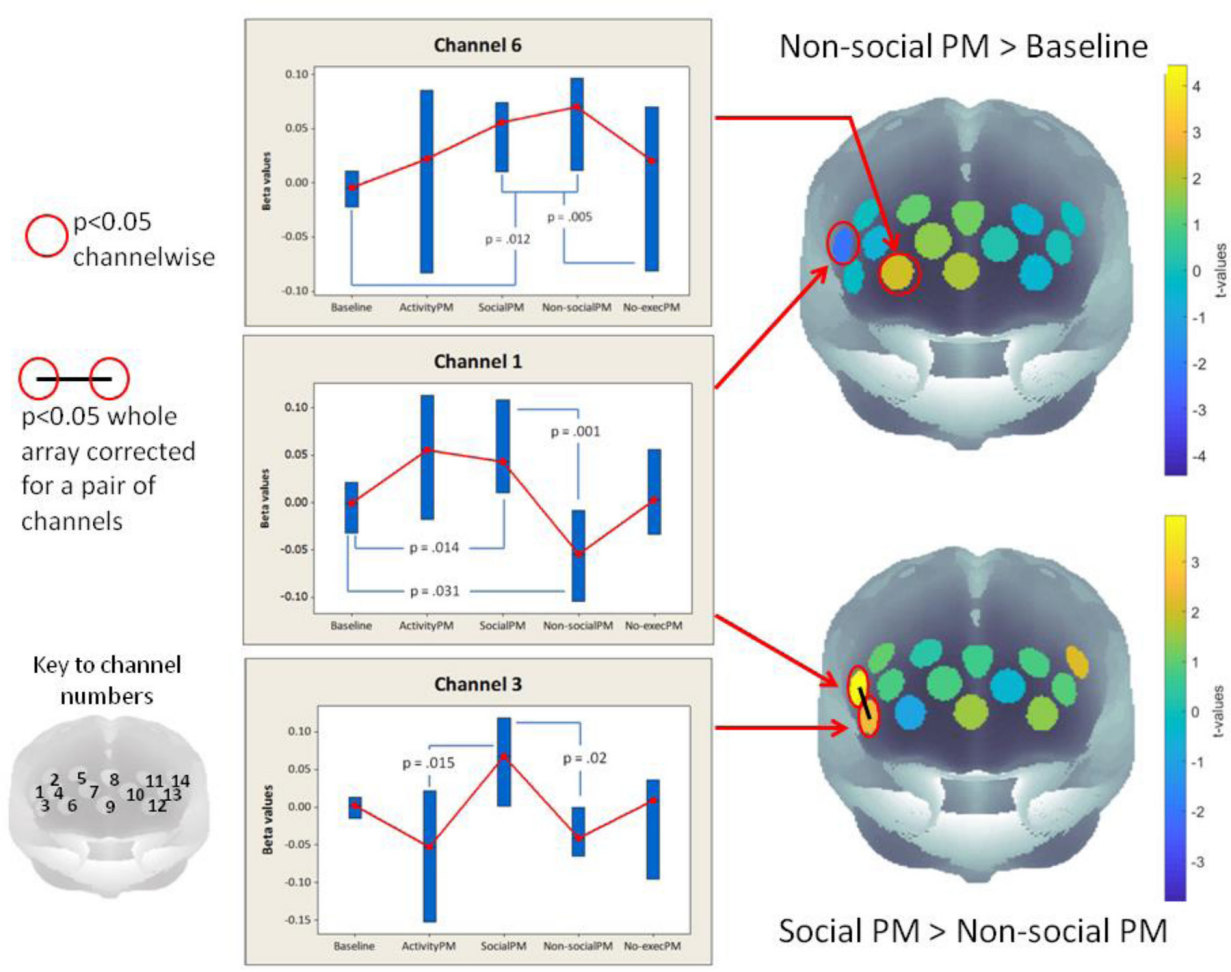
Non-social event-based prospective memory versus baseline, and versus social event-based prospective memory. Channels 1 and 3 (right dorsolateral PFC and inferior frontal gyrus, BA 46 & BA 45/47) were significantly higher in the social prospective memory condition than the non-social PM condition (corrected for multiple comparisons using a “two adjacent channels” criterion, see text for details). Boxes in the graphs are 95% CI for the median, shown as red circles, with uncorrected statistics for conditions contrast given in the graphs. (For interpretation of the references to color in this figure legend, the reader is referred to the web version of this article.).

For the second, exploratory stage, we considered all channels and corrected for multiple comparisons, as described above. The most critical comparisons are between the two event-based conditions and the baseline, and between the two event-based PM conditions themselves (i.e., social vs. non-social PM). In line with our a priori results, we find that the activation of channels 6, 8 and 9 in the Social PM > Baseline contrast meets the array-level threshold (see Figs. 3 and 4). For the non-social condition the channel activations for the non-social condition *compared to baseline* were in general less strong overall throughout PFC than found in the comparison between baseline and the social condition, and although channel 6 (approximating BA 10 and 11) showed a significant activation increase at an uncorrected level, this comparison did not yield activations that survived our multiple comparisons criterion of two adjacent significant channels.

However, there was a strong effect of the social vs. non-social comparison for two adjacent channels covering right dorsolateral and inferior lateral PFC (channel 1, BA45 approximate MNI location 54, 38, 12, and channel 3, BA 46: 48, 54, -2) which survived our strict correction for multiple comparisons (see Fig. 4). Light shielding issues meant that we could not investigate the homologous lateral regions within the left hemisphere, but channel 14 (left dorsolateral PFC, BA45/46) is the nearest left hemisphere location for which we have data, and interestingly, this channel showed significantly higher activation at an uncorrected level for the social prospective memory conditions compared to baseline (t = -2.11 p = .049 uncorrected), and also a higher activation for the social condition compared to the non-social (t = -2.19, p = .042, uncorrected). Finally, comparing activity-based PM with the overall baseline, there were no regions of PFC showing a significantly different level of activity. There were also no reliable activations when comparing PM events to null events (PE > NE).

### 3.3 Recovered functional events: spatial and temporal characteristics

**Fig. 5:**
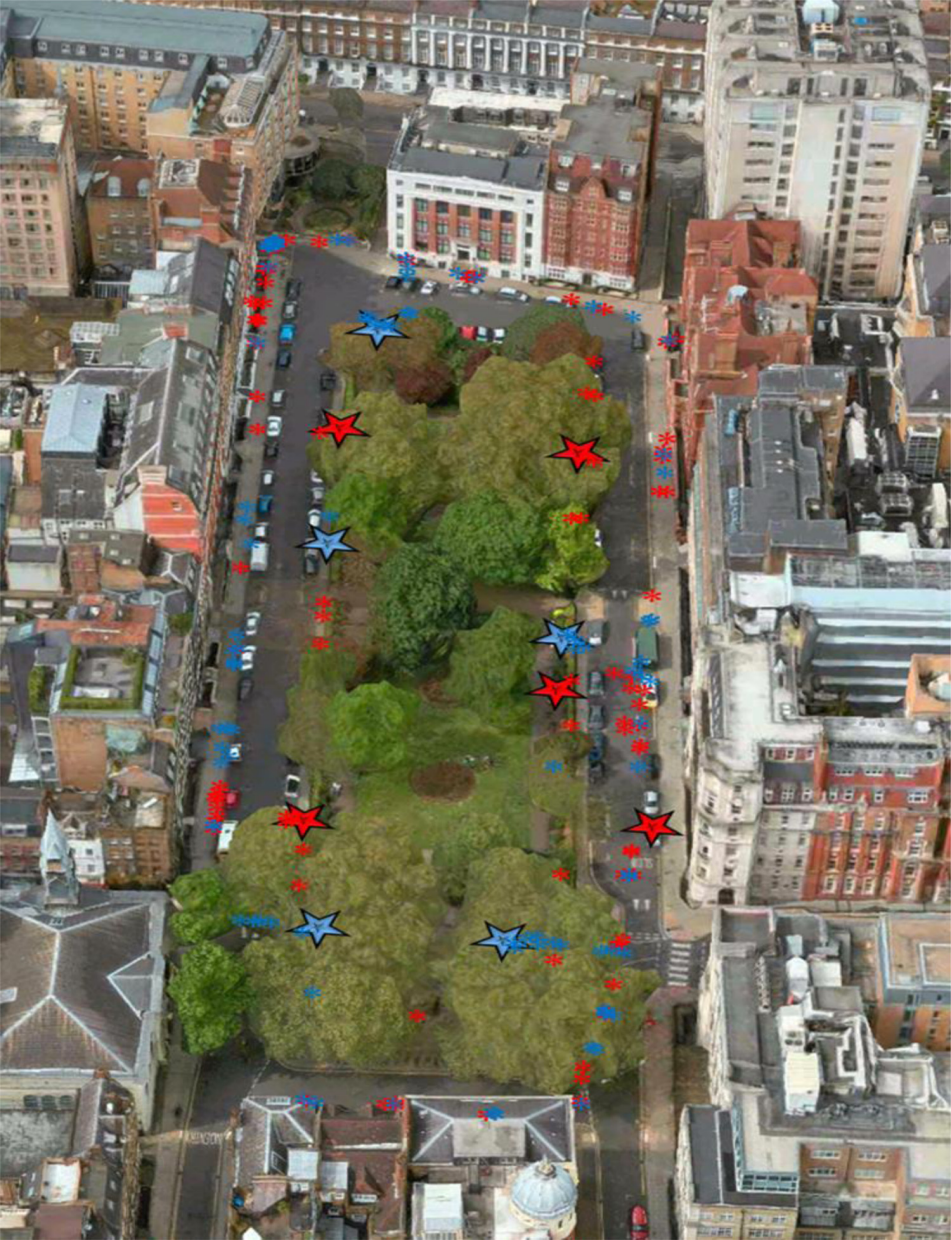
A map of Queen Square showing the spatial locations of rostral PFC functional events. Large blue and red stars indicate the location of the social and non-social PM cues respectively, and the blue and red asterisks represent the locations of participants when social and non-social functional haemodynamic events occurred, respectively. The peaks in activity in rostral prefrontal cortex tend to occur when the participants were close to the prospective memory cues (either the confederate or a parking meter), especially a few seconds before the intention was executed. (For interpretation of the references to color in this figure legend, the reader is referred to the web version of this article.).

Regarding neural event location, for each onset timing of the detected functional events, the participant's physical location at that moment was marked on a map using video data that was synced with the fNIRS time-series. Condition-specific functional neural events tended to aggregate around task-related moments, as detected by the AIDE procedure. In the social PM condition, events largely occurred where the intention to interact with a confederate (i.e., the fist-bump) was executed, and also just before this behaviour, when participants might be expected perhaps to have spontaneously retrieved this intention upon seeing the confederate. Similarly neural events detected in the non-social PM condition largely occurred where the intention to “fist-bump” a parking meter was executed, and just before this behaviour, when participants might be expected to be spontaneously retrieving this intention upon seeing the parking meter (Fig. 5).

**Fig. 6:**
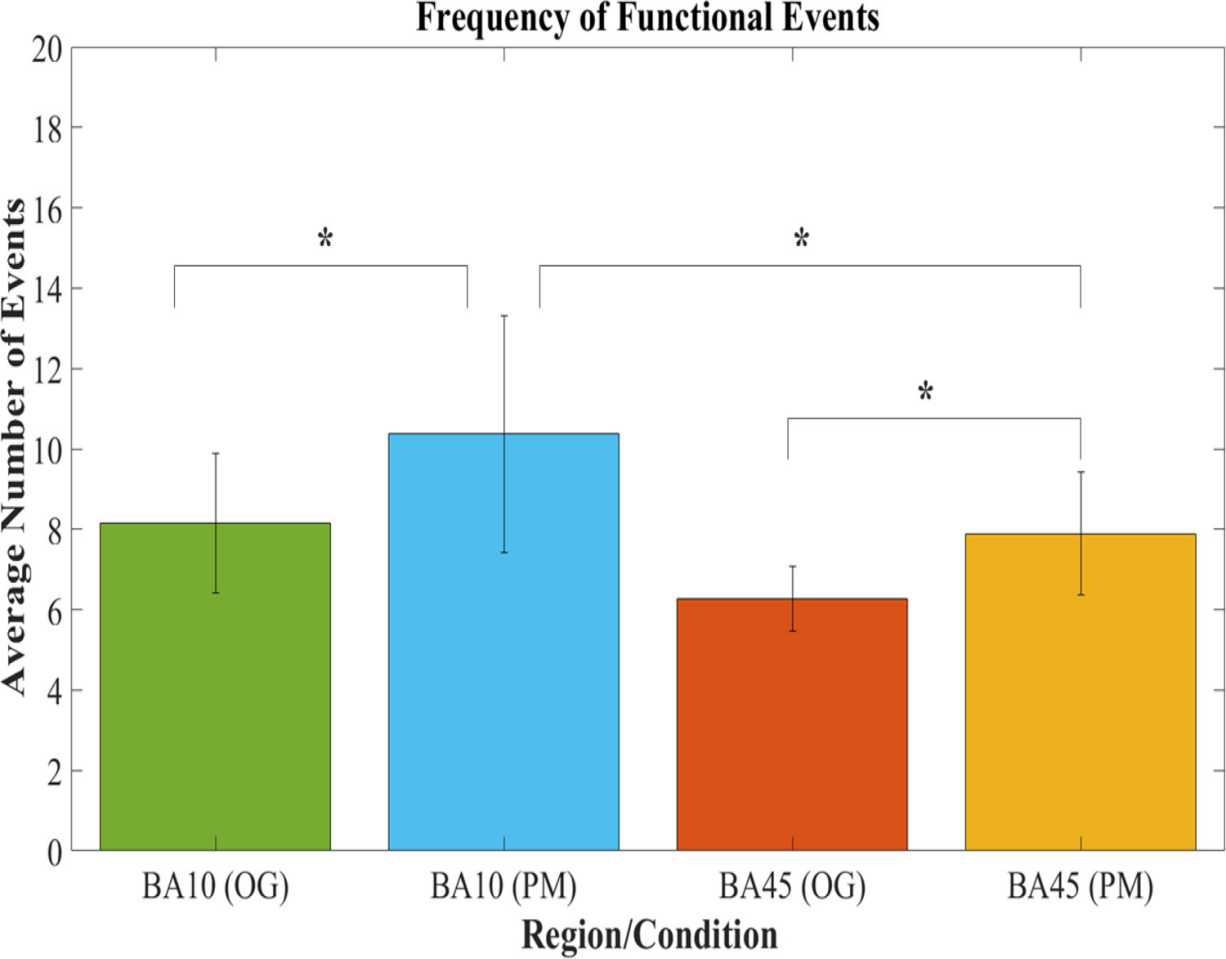
Comparisons of the average group frequencies of functional events detected in rostral PFC during the OG (*green*) and PM (*blue*) tasks, and in lateral PFC during the OG (*orange*) and PM (*yellow*) tasks. The frequency of functional events increased most in rostral PFC during the PM tasks. An asterisk indicates a p-value ≤ .01. (For interpretation of the references to color in this figure legend, the reader is referred to the web version of this article.)

As regards event frequency, functional neural events as detected by AIDE were more frequent in rostral PFC compared to lateral PFC during both the PM and OG tasks (Fig. 6). Moreover, the average number of functional events in the sample were more frequent within rostral PFC during the prospective PM task, collapsing across social and non-social prospective memory conditions, than during the OG task conditions, *t*(18) = 3.84, *p* = .001, *d_s_* = .94, 95% CI [1.0, 3.42].

**Fig. 7:**
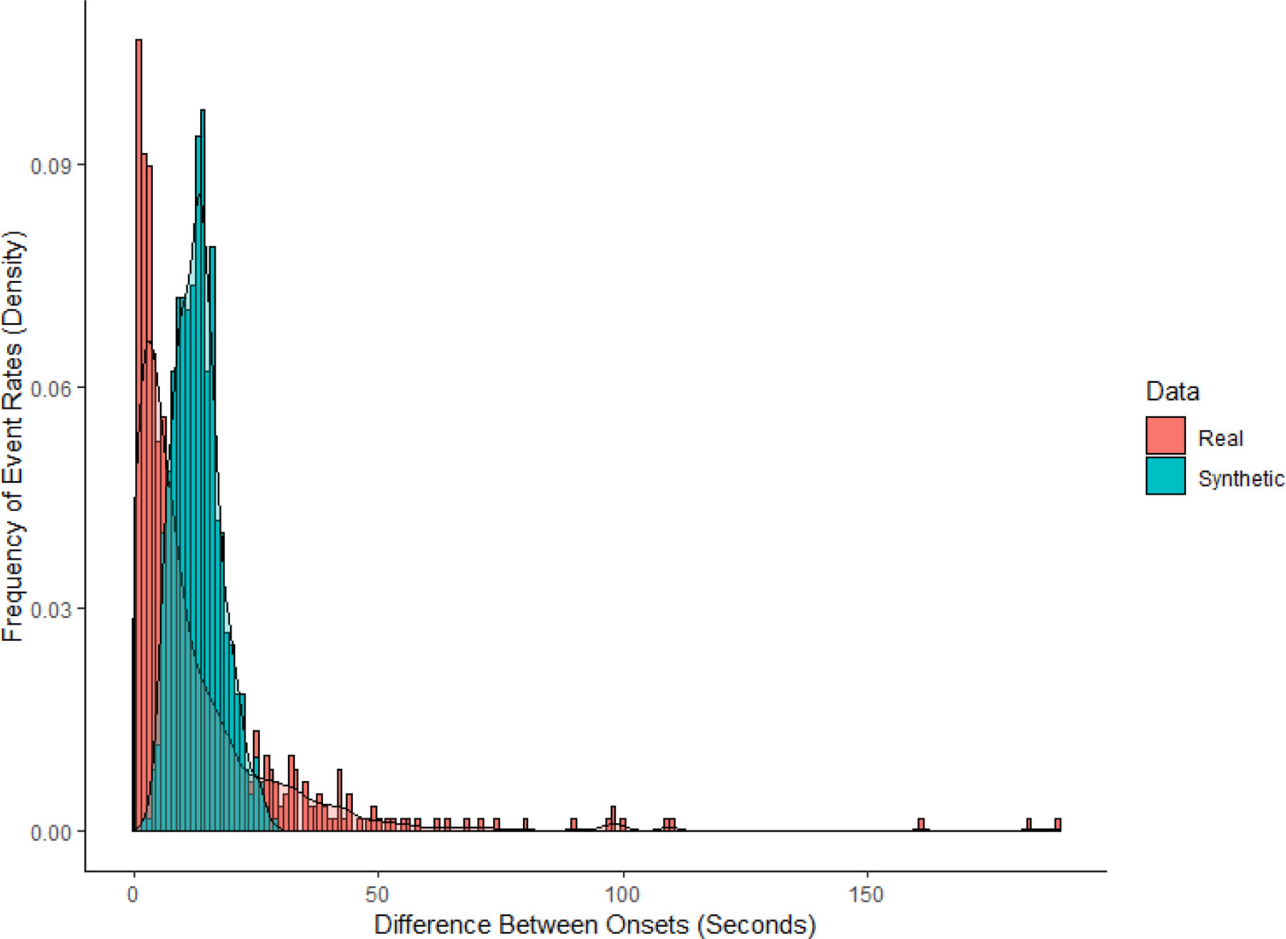
Group comparison of the real (*red*) and synthetic, Poisson (*blue*) distributions of the variation in functional event onset timings collapsed across tasks and subregions (rostral and lateral PFC). The real data significantly differed from the synthetic distribution: The real functional event onsets were too variable to be described by a random process, in that the nature of the task sometimes elicited a fast burst of functional events and sometimes slower, more periodic ones. (For interpretation of the references to color in this figure legend, the reader is referred to the web version of this article.)

With respect to event rate, if the detected functional events in these conditions were not task-related and, therefore, explicable by some random process (e.g., Poisson variable; Gabbiani and Cox, 2010), then there should be no significant difference between the timings between event onsets (i.e., temporal variation) and synthetic Poisson distributions; however, there was such a difference between the real and synthetic data, *U* = 114004, *p* < .001, *d* = 0.64 (*Z* = 10.57) (Fig. 7).

**Fig. 8:**
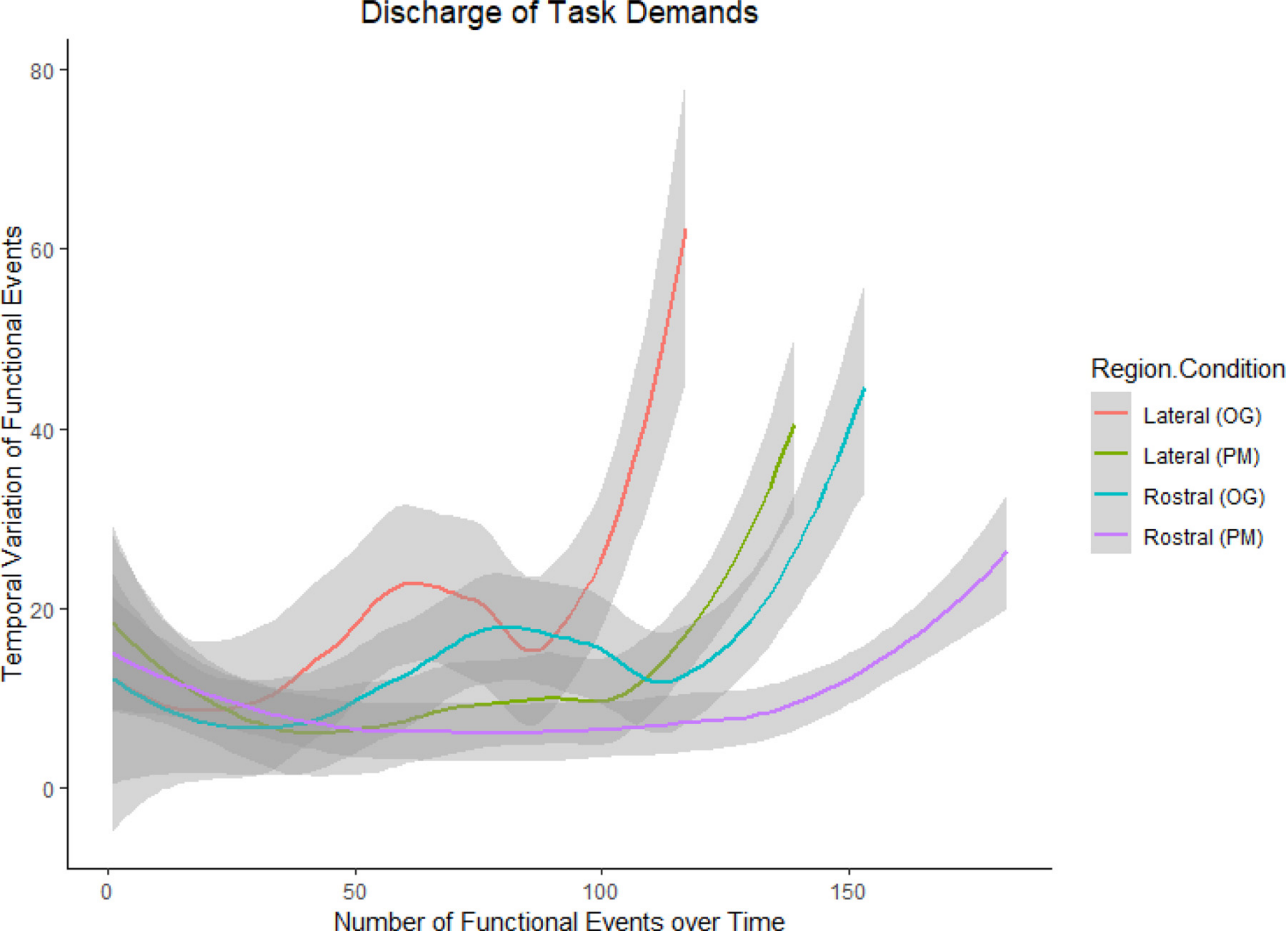
Group variations in functional event onset timings. Event rates for rostral PFC during the OG (*blue*) and PM (*magenta*) tasks, and lateral PFC during the OG (*orange*) and PM (green) tasks, fluctuated throughout their respective blocks, but each task showed a slowing in event rate as the task neared completion, with rostral PFC in the PM condition showing the least sharp drop in functional events. This is what would be expected of a subregion that is critical to a task (i.e., consistently and dynamically engaged throughout it), of a subregion that shows a high event frequency (Fig. 6). (For interpretation of the references to color in this figure legend, the reader is referred to the web version of this article.)

Interestingly, a marked decrease in event rate was observed (i.e., longer time periods between functional events) across every condition and ROI near the end of each task (Fig. 8). This growing drop in the incidence of functional events is consistent with the idea that the cognitive resource requirements across rostral and lateral PFC should decline near the end of carrying out the demands of a given task; indeed, there is a moment shared by each participant where they will realise that there will be no more retrieval cues (i.e., parking meters or confederates) between where one is currently located and the end of the test, because one can easily see the endpoint ahead.

## 4 Discussion

Our data reveals, for the first time to our knowledge, the role of PFC in a naturalistic prospective memory task beyond the confines of the laboratory and confirms that the neural phenomena under examination are sufficiently robust for detection using mobile fNIRS. Considering first the condition contrasts, we found that performance of the event-based prospective memory conditions (which consisted of performing an ongoing task plus the addition of a delayed intention demand), relative to baseline conditions that did not involve an intention, was associated with increased activation in a substantial region of rostral PFC (BA 10), stretching from medial to lateral aspects within the right hemisphere. Moreover, when contrasting social and non-social prospective memory conditions against each other, we found activation in lateral PFC (BA 45 and 46) within the right hemisphere. In addition, using the “brain-first” approach developed for fNIRS by this research group (Pinti et al., 2017) we found that the functional (haemodynamic) events detected in the social and non-social PM conditions tended to occur when participants were physically near to the targets of these conditions on the street. These events occurred most frequently in rostral PFC during the prospective memory (PM) conditions, complementing the findings of the blocked-design. We discuss first the methodological issues associated with our novel approach and then the implications of the neuroimaging results for our understanding of the cognitive neuroscience of prospective memory.

### 4.1 Why study neurocognition outside the lab?

There are two principal reasons why we have examined our hypothesis in the way that we have, i.e., outside the lab, in a naturalistic situation with freely ambulant participants who are deciding for themselves when, where and what they will do. The first reason is that the prefrontal cortex, which we are studying here, is widely accepted to be involved in voluntary control and organisation of behaviour, especially over long time periods (for review see Burgess and Wu, 2013). The more usual lab-based neuroimaging methods (e.g., fMRI and PET) are highly constrained in the activities that can be studied, because participants need to be supine, usually with the body and head immobile. Thus, voluntary movement is highly restricted. Furthermore, the computer-based and highly structured nature of most typical neuroimaging experiments means that the frontal lobe processes that are involved in longer-term behavioural organisation, and in dealing with open-ended situations and self-initiated behaviour, are tough to study in realistic ways using those experimental formats (for more discussion on this point see Pinti et al., 2018). The second reason why we have examined our hypothesis that there will be a neural signature of the prosocial intention superiority effect by using mobile, wearable fNIRS, is that we can be more sure that a task is measuring social processing if it involves direct interaction with another person, including meaningful physical contact of the kind that occurs every day in human interactions. In this way, not only is the ecological validity of the task stronger, but also the construct validity.

Here, we consider first why fNIRS is particularly well-suited to the study of prefrontal cortex in real-world contexts, summarising both the advantages and practical limitations of the methods. Then we consider the question of how we can design and implement cognitive experiments in unconstrained contexts and explore how such data should best be analysed.

As a method for human neuroimaging, fNIRS is perhaps unique in terms of suitability for the study of neural processes in naturalistic contexts with freely moving participants. Optically pumped magnetometers (MEG) allow for more physical movement than fMRI, but the participant needs to be kept in a non-naturalistic shielded lab environment, so the freedom and ease of movement cannot match fNIRS. EEG allows for considerably more freedom of movement than fMRI or MEG, but remains prone to motion artefacts. Furthermore, the poor spatial resolution and low signal-to-noise ratio of EEG puts considerable constraints upon experimental design (e.g., requiring many repetitions of the same event) and therefore the types of behaviour that can be studied. This is particularly an issue when dealing with a cognitive system such as the frontal lobes, which is intimately bound up with dealing with novelty (Burgess, 1997). So, while these neuroimaging methods (fMRI, PET, MEG, EEG; for discussion of the relationships between fMRI and fNIRS findings see e.g. Cui et al., 2011; Eggebrecht et al., 2012; Noah et al., 2015) have been remarkably successful for exploring the neuroanatomical substrates of much of human cognition, it is difficult with them to study behaviour: (a) that occurs in “naturalistic” environments, (b) when the participants are freely moving around, and (c) when there will be a limited number of instances of certain events or conditions. Yet much of typical human social behaviour, especially that which involves the prefrontal cortex, has these three characteristics.

Accordingly, here we exploited the advantages of wireless and wearable fNIRS for studying brain activity in naturalistic environments to collect measurements of neural activity outside the lab, while participants were engaged in social behaviour (e.g., remembering to watch out for, and greet, another person) like those in everyday life. In our previous study (Pinti et al., 2015), we demonstrated the feasibility of this new generation of wireless, wearable fNIRS devices for naturalistic applications. More precisely, the WOT system we used is wearable, wireless and lightweight (portable box: 650g; headset: 700g). In naturalistic conditions, we found it to be stable and tolerant to motion artefacts, with most optodes well-shielded from sunlight by a shading cap (only the optode orientation for channels 15 and 16 was problematic).

There were some practical issues that we had to consider in implementing the study. We had to deal with inclement weather (the fNIRS unit was not waterproof), and we unfortunately lost signal from two of the channels due to poor light shielding. This loss was not foreseeable since no-one had attempted an experiment like this before (data collection started in winter 2014), in this location and with this equipment. But for this reason, we are cautious about claims of hemispheric differences in lateral PFC. In particular, this relates to the lateral PFC signal changes noted in the social PM > non-social PM contrast. Some of these occurred in the right hemisphere channels for which there is no left hemisphere equivalent in this experiment.

In addition, it is important to mention that fNIRS signals can be corrupted by physiological changes of non-neural origin (e.g., heart rate, breathing rate, blood pressure) caused by systemic activity originating both at the intra- and extra-cerebral compartments of the head (Tachtsidis and Scholkmann, 2016). These act as confounding factors and can lead to false positives and false negatives at the statistical inference stage. Some of the ways to approach and rectify this issue are (i) to monitor physiological parameters (e.g., heart rate, breathing rate) alongside fNIRS measurements and remove their effect from fNIRS signals; (ii) have baseline, control conditions that allow to subtract out systemic brain hemodynamic responses from the functional task; and (iii) use both HbO_2_ and HHb in the statistical analysis framework (Tachtsidis and Scholkmann, 2016). In this study we employed the CBSI method that creates an activation signal based on maximising the anti-correlation between HbO_2_ and HHb, which is expected to occur during brain functional activation (Cui et al., 2010). In addition, our experimental protocol design included control periods that involved walking and stopping; also, the functional contrast for the inference of activation between conditions had the same systemic baseline levels. In addition, although this is the extent to which the present study addressed the issue of systemic confounds, variables such as extra-cerebral blood flow (e.g., skin-blood-flow) might plausibly still be an issue (e.g., Takahashi et al., 2011) and should be further accounted for by using techniques such as short-separation channels and attenuation algorithms (Keshmiri et al., 2019; Sato et al., 2016). See Yücel and colleagues (2021) for further review of quality control measures.

Beyond the practicalities of using an fNIRS device, there are also many important experimental design constraints when neuroimaging in so-called “ecologically valid”, naturalistic situations (or, more correctly, those with high “representativeness”, Burgess et al., 2006a) where the participant is free to behave as they want to and when they want to. In particular, we can examine how our naturalistic paradigm differs from traditional cognitive experiments carried out in the lab. Perhaps the most important difference is that, in naturalistic studies, it is rarely possible to control perfectly the onset timings of stimulus presentation. With greater ecological validity comes greater situational complexity: No two participants will experience the same pattern of ‘stimulus presentation’, despite great efforts on the part of the researchers to control key events during the naturalistic task performance. That is, there is likely to be substantial inter-individual variability in stimulus exposure. This contrasts with typical lab-based neuroimaging experiments when the appearance of events or targets can be determined with millisecond precision. Similarly, the duration of events is typically well-controlled in lab studies but can be very variable in natural environments. This difficulty of contriving stimulus onsets and durations in real-world situations can be referred to as the ‘stimulus-design problem’.

These timing factors might need to be determined in retrospect rather than in advance to address this issue. That is, rather than endeavouring to account for all the complexities of the real world in an experimental design, a more advantageous approach might be to recover the experimental design from the real world by adopting an *a posteriori*, brain-first approach. So, identifying functional event onsets and durations within a naturalistic environment is a major challenge, and towards that goal we have recently described an approach that uses the observed haemodynamic response data to identify the timing of these events (Pinti et al., 2017). The findings that evaluated its accuracy in terms of event location, frequency, and rate suggested that this technique addresses the stimulus-design problem. However, some computer mediation might be required in future research to further validate AIDE. For example, future research not involving aspects of navigation—and, therefore, spatial distributions of events—might test AIDE by assessing event frequency between subregions of interest during tasks derived from traditional experimental psychology.

In addition, the “open-endedness” (or “ill-structured” nature) of these (and many other) everyday situations contrasts starkly with the “well-structured” nature of typical lab-based neuroimaging experimental designs. In other words, there are typically many ways for the participant to achieve their goal and it is up to them to choose a particular approach. This can affect simple factors like gaze: the direction of the participants’ gaze and visual attention during the task is typically controlled in lab studies with, for example, a pre-stimulus fixation and an environment with minimal distractions. This is very different from typical everyday life situations, where a participant is free to attend to whatever they choose in a complex, dynamic and interesting environment. But open-endedness also acts as a bigger timescale, as participants are free to choose where to move in the environment and how to engage in the overall task demands.

This is an important issue when studying cognition supported by prefrontal cortical structures since rostral PFC (the largest subsection of PFC, and one of the largest cytoarchitectonic subregions of the cortex, see Bludau et al., 2014) in particular seems to support mental processing that is particularly required in dealing with open-ended situations. For instance, it has been known for some time that neurological patients with rostral PFC lesions may show problems with dealing with open-ended situations, even ones that seem “easy” to healthy controls, and even when their performance on “difficult” but well-structured tests such as IQ tests is excellent (see Burgess and Wu, 2013, for review). In these ways, although neuroimaging in naturalistic environments makes special experimental demands, it also offers an excellent opportunity to explore certain cognitive processes which can be a challenge to study within the necessarily well-structured neuroimaging lab environment. It may be this increased construct validity, in regard to these particular processes, that has resulted in strong effects in our study despite using a highly unconstrained (by lab standards) testing situation. However, this possibility remains to be tested directly.

### 4.2 Cognitive interpretation of our results: the role of PFC in prospective memory

Our study yielded three particularly noteworthy results that illuminate the role of prefrontal cortex (PFC) in prospective memory. The first concerns activations in rostral PFC (BA 10). Performing the event-based PM tasks, compared to baseline, resulted in activation in rostral PFC (BA 10), mirroring the patterns found in previous fMRI and PET studies of laboratory event-based PM tasks. It is interesting to note therefore that fNIRS seems sensitive enough to these sorts of cognitive manipulations to detect reliable PFC activations even in “real-world” naturalistic situations. In addition, we found that activation within rostral PFC (BA 10) was more extensive for the social prospective memory condition than the non-social one (see Figs. 3 and 4). To our knowledge, this has never before been shown. However, the social/non-social difference in patterns of activation went beyond relative increases alone. Our second principal finding was that when social and non-social prospective memory conditions were contrasted directly, we found significant activation increases in the social condition in right lateral PFC (BA 45 and 46). There was also an increase within the lateral left hemisphere, although this was only apparent at an uncorrected level. The third principal finding emerged out of the AIDE analysis of neural events, which examined where participants were on the street, and what they were doing, when peaks of activation were detected. We found that the distribution, frequency, and rate of the PFC functional events that were detected from the observed fNIRS data reflected task-related information processing, and therefore the spatial position of the participant on the street, in the real-world environment. We discuss the interpretation of each in turn.

### 4.3 Rostral PFC (BA 10) activations

The rostro-medial PFC activations that occurred in the event-related PM conditions relative to baseline were significant only for the social PM condition. However, the medial activation in the non-social condition was (i) not significantly different from that found in the social condition, (ii) was higher than in the baseline, and (iii) both social and non-social condition activations in medial rostral PFC when considered together were significantly higher than in the no-execution PM condition. In other words, the medial rostral PFC pattern of activation was similar for the non-social condition, but less marked. One possible interpretation of this pattern relates to a key question for the cognitive science of prospective memory, which is how a social intention superiority effect might occur. Behaviourally, participants in this experiment did not show significantly better performance in the social PM conditions compared to the non-social one. However, the pattern of differences was in the expected direction (better performance in the social condition, accompanied by an “intention cost” (Burgess et al., 2003) upon the ongoing task), and as outlined above, there were significant brain activation differences. Accordingly, let us suppose that there might be two possible neural correspondences for a social intention superiority effect. The first would be where the patterns of activation are similar in social intention situations as non-social ones, but that the activation is stronger (i.e., a “same but stronger” account). The second simple possibility is that the patterns of activation are different in the social vs. non-social intention conditions (i.e., the “different not stronger” account). The medial rostral activations here (principally, BA 10; channel 9, and to a lesser extent channel 8) appear to be an example of the “same but stronger” account. In other words, this may not be processing that is uniquely “social” in nature, but is utilised to a greater degree when social intentions are involved.

There are a number of accounts of the role of rostral PFC (BA 10) in PM (Beck et al., 2014), but probably the account most thoroughly investigated in neuroimaging studies to date is the gateway hypothesis of rostral PFC function proposed by Burgess and colleagues (e.g., Burgess et al., 2005, 2006a, 2006b, 2007; Burgess and Wu, 2013; Dumontheil et al., 2010; Gilbert et al., 2005 ). Over the past 15 years or so, this hypothesis, which assigns a particular attentional role to rostral PFC, has been tested by other groups (e.g., Henseler et al., 2011) and applied to the patterns of activation in prospective memory studies (e.g., Barban et al., 2014). This hypothesis suggests that rostral PFC supports an attentional system that serves to bias the direction of attending behaviour towards either stimulus-oriented attending (i.e., attending to stimuli in the external world) or towards stimulus-independent attending (i.e., attending to the thoughts in your head) in situations where novel degrees of control are required. Burgess et al. (2001, 2003) suggested that perhaps prospective memory requires such an attentional “gateway” because maintaining and then activating a delayed intention involves interplay between attending to the environment (to spot the PM cue) and stimulus-independent thought (to maintain the intention). (See also Okuda et al., 2007, 2011). The role of this attentional system in prospective memory has been confirmed both by direct empirical investigation and by meta-analysis. The first direct empirical test was by the Burgess group itself (Benoit et al., 2012), with the conclusions of that study broadly replicated by another group (Barban et al., 2014), who concluded that “these results are in agreement with the gateway hypothesis: during a PM task medBA10 biases attention toward external salient stimuli, SO attending, while latBA10 biases attention toward internal mnemonic representations, SI attending” (p. 203). More recently, Cona et al. (2015) reported that their meta-analysis of neuroimaging prospective memory experiments “confirmed that intention maintenance was consistently associated with lateral BA 10 activation coupled with medial BA10 deactivation… This finding supports the Gateway Hypothesis, according to which the aPFC is involved in the biasing of attention between external stimuli… and internal thought processes” (p. 30).

On the basis of this agreement, then, the most obvious starting point for an interpretation of the rostral PFC activations in this experiment relates to the gateway hypothesis of rostral PFC function as applied to prospective memory. This would hypothesize for instance that the PM conditions, and the social one in particular, require or induce an increased amount of switching between attending to external stimuli and the thoughts in one's head. This might happen if one was visually searching for the confederate in the social conditions while trying to remember the information that needs to be collected on the street. It might be worth pointing out in this respect that one difference between the social and non-social conditions was that the confederate moved location, whereas parking meters were stationary. A related visual attention type hypothesis has recently been involved for BA10 activations during a social neuroscience experiment that involved face-to-face deception of another individual (Pinti et al., 2021). However, in the absence of further evidence, this must remain a working hypothesis only.

### 4.4 Lateral PFC activations

The second finding, a significant lateral PFC activation difference (BA46/45) in the social vs. non-social PM contrast, appears to be an example of the “different not stronger” account outlined above. This invites the question of what is different about the social condition compared with the non-social one. On present evidence, there are a number of possibilities in particular that might be considered. However, all of these might lead to one super-ordinate level of explanation that arises from prospective memory theorising.

So, for instance, one might argue that social interaction is more rewarding, and so this is the source of the higher lateral PFC activation in the social PM condition. In support, McCauley et al. (2009) have shown a relation between increased reward and performance on PM tasks; social interaction may be a primary reinforcer that is even stronger. However, it may not simply be a matter of how important the PM task is being appraised, but an interaction between this and the increased attentional monitoring demands that follows from this change in importance (see e.g., Einstein et al., 2005; Kliegel et al., 2001, 2004). On this kind of account, there is increased self-monitoring in the social condition (i.e., because you are more self-conscious if you think that the confederate is watching or expecting you). This may invoke self-monitoring and error-monitoring processing, which are forms of mental activity that have been associated with right PFC function (e.g., Harty et al., 2014). Other plausible explanatory possibilities include, for instance, the increased sustained attentional demands invoked by conditions a person may appraise as more personally significant: Sustained attention tasks have been shown to activate similar brain regions (e.g., BA 46 and 47) in fMRI studies (e.g., Grahn and Manly, 2012). Another account holds that dorsolateral PFC subregions may be involved with assessing social outcomes of an observed individual's actions (Weissman et al., 2008).

But perhaps the greatest link with work outside of the field of pure prospective memory research might be made with rehearsal or “working memory” processing. For instance, D'Esposito and colleagues (e.g., D'Esposito et al., 1998) have implicated area 46 and nearby regions (i.e., the region that was more highly activated in the social vs. non-social condition contrast) as involved when people are actively maintaining information over a delay period (e.g., rehearsal components of working memory; see e.g., Basso et al., 2010). Indeed, the association between certain working memory functions and lateral aspects of PFC seems secure, on the basis of both lesion and neuroimaging studies (e.g., Muller and Knight, 2005; Owen et al., 2005; Petrides, 2005). On these grounds, perhaps certain aspects of the social prospective memory condition (e.g., being observed, and/or the perceived significance of the social interaction with the confederate) alter the way that the participant addresses the task, increasing active intention rehearsal (the controlled processing component related to working memory) relative to the non-social condition. This might occur, for example, as a reaction to the increased monitoring of the environment. In other words, if the participant is trying to remember the information they are gathering (the ongoing task) whilst also monitoring their environment more, this may require concomitant increased use of a rehearsal strategy. It is not however possible on the current evidence to decide between these alternative scientific narratives. In fact, it may be that these accounts intersect at an attentional level; in other words, perhaps critical information processing aspects of what we might label “assessing social outcomes” and “self-monitoring” utilise a shared attentional control process.

Returning to the rostral PFC activation in the event-based PM conditions, one issue that remains to be addressed is why they evoked activation increases in medial rostral PFC (BA 10) rather than the more usual pattern of decrease that occurs while maintaining an intention (for review see Burgess et al., 2011). One possibility that would deserve further investigation is that the experimental PM blocks probably required three demanding processes*:* (a) maintaining a thought in your head (e.g., bearing in mind that you have to fist-bump the confederate or parking meter while doing the ongoing task) (b) attending more closely to the test environment to detect the PM targets than the ongoing task alone requires; and (c) perhaps rapid switching between a and b. In other words, these two potentially dissociable attending modes cannot be dissociated using the method utilised here. It is also worth noting that, because the above discussed findings were mostly from females, it is unclear whether they would be consistent with future observations that are drawn from a more equal distribution of sex. It might be possible that, for example, resource consumption in the PFC would increase as the proportion of males becomes larger (e.g., Li et al., 2010; Weiss et al., 2003); or there could be sex-specific demands of processing prospective memories that are material specific (e.g., Haut and Barch, 2006), such as those which are social in nature.

### 4.5 Temporal signatures of PFC activations

It is interesting to note that our contrasts between activation at the specific time of responding to the PM targets (compared to activation at other times) did not reveal any robust activation within prefrontal cortex (PFC). This is perhaps to be expected for two reasons. First, identification of PM targets is most associated with activation deep within, or outside prefrontal cortex, especially anterior cingulate, and several regions within temporal, parietal, and occipital lobes (BA 40/39/18/19; Simons et al., 2006), with additional transient activation of the middle temporal gyrus relating to PM targets (BA21/37; Reynolds et al., 2009). Our fNIRS headset however was only measuring from the surface of PFC. Second, there is the difficulty arising from determining the time of cognitive events from behaviour recorded on video. That is, from our video of the task we coded the time when the participant performed a PM action (e.g., fist-bump) but could not reliably detect the time at which the participant first detected a target (e.g., saw the confederate on the other side of the street). The latter is the point at which the delayed intention becomes an active intention and could occur up to 30 seconds before the PM action itself (e.g., if there was traffic and the participant waited to cross the road) or just a few seconds before (e.g., if there was no traffic). The addition of eye-tracking data from a mobile eye-tracker, as well as GPS tracking, might have improved our ability to detect the relevant cognitive events in this task, but as people can gaze at a stimulus without detecting it (e.g., Smith et al., 2012) this would still not be a guaranteed method for deciding at what time point in the study did the participant detect the PM target.

Fortunately, the application of a brain-first approach (i.e., AIDE) to recovering stimulus designs from ecological settings largely addresses this issue and, importantly, augments the explanatory power of findings deriving from blocked-designs by taking advantage of the temporal resolution of fNIRS (<1Hz) to improve our spatial understanding of the data in two senses: First, we were able to map out where participants were within the testing environment during the onset of significant functional events and second, we were able to map the brain regions that generated these activations. This revealed that functional events from the social and non-social PM conditions tended to occur in non-random locations, particularly where participants appeared to see and then approach a target of the respective condition. But Fig. 5 also shows other functional events occurring around moments of intention execution as well as perhaps those related to monitoring for intentional cues. The general aggregations of condition-specific events suggest at the very least that these events were indexing task-related processing. This was supported by the finding that the greatest frequency of functional events was detected in rostral PFC (BA 10) in the PM conditions compared to other conditions and regions. The PM conditions elicited a higher frequency of functional events in both rostral and lateral PFC compared to the ongoing ones, and event frequency across PM conditions was greatest in rostral PFC compared to lateral PFC (Fig. 6). This is consistent with what one would expect of an attentional subsystem localized in rostral PFC that is required to switch frequently and dynamically between attentional modes during an event-based PM task. Interestingly, comparing the functional event rates (i.e., temporal variation between events) across the PFC regions of interest to synthetic event rate distributions showed that these rates were too variable to be random (i.e., there were periods of time across the group where the incidence of these events would speed up and then slow down, and then speed up again, and so forth) (Fig. 7). This provided further support for the validity of the use of the AIDE methodological procedure on naturalistic tasks.

In addition, a chance finding was the marked and sustained drops in the incidence of these neural events near the end of each task (Fig. 8). The notion of a discharge of intention-related processing towards the completion of a task has a long history going back to Kurt Lewin, with his characterisation of a release of “goal tension” as intentions are completed, with the Zeigarnik Effect (the tendency to remember unfinished or interrupted tasks better than completed tasks) as a behavioural consequence (Lewin, 1935). This accidental finding from the use of AIDE, and potential explanation, raises an intriguing line of inquiry, clearly requiring further investigation. New studies could also focus on determining if brain-first approaches can be improved to involve the classification of detected functional events into particular operations of information-processing systems (e.g., social intention creation, maintenance, retrieval, & execution). That is, distinguishing between event types would potentially improve the specificity of brain-first approaches, such as in a way that is similar to multi-voxel pattern classification used on fMRI data (Chow et al., 2018).

## 5 Conclusion

There are two main conclusions from this study. The first is methodological. We have shown that it is possible, using fNIRS, to measure prefrontal cortical activity linked to complex mental activity while adult participants are engaging in a “real-world” naturalistic prospective memory task, conducted outdoors, in freely moving participants. The experimental conditions were varied, and participants were free to choose where to go (within certain geographical parameters), and when to go. No special preparation of the environment was undertaken: This was a busy London UK street, with all the usual happenings occurring around the participants that occur in inner city centres, and although the participants were instructed about the task just beforehand, they were not specially trained in any way, and were selected from a typical UK university student population. A complementary analysis method (i.e., AIDE) also revealed when and where PFC functional events occurred in this environment.

The second main conclusion is a cognitive neuroscience one. We have revealed activation differences during the social prospective memory condition relative to the non-social one. Medial and lateral rostral PFC (area 10, frontopolar cortex) regions showed greater activation during both the social and non-social PM conditions compared to baseline, and also compared to a condition where the PM targets (the confederate or the parking meters) were present but the participant did not have to maintain a delayed intention. But activation in lateral PFC regions (BA 45 and 46), especially on the right, was significantly higher during the social condition compared to the non-social one. One interpretation of this pattern of activation might be that it is a neural signature related to the “social intention superiority effect”, which is the tendency to rate as more important, and carry out more effectively, delayed intentions that have a social component compared with ones that do not.

In terms of the information processing models of prospective memory outline in the Introduction, the results here are less in agreement with the Spontaneous Retrieval Theory (e.g., McDaniel et al., 2004) of prospective memory, since there did appear to be regularities in the temporal patterns of activation and the locations where they occurred. The Preparatory Attentional and Memory (PAM) processes theory (e.g., Smith, 2003) predicted the activations would be likely to be seen irrespective of the PM cues, temporally and spatially. This also does not seem a particularly good fit to the data presented here (although of course in other situations these accounts might fare much better) since the activation patterns seemed to be clustered around the PM cues, although often in anticipation of them. Instead, the data from this experiment is most consistent with the predictions from the Multiprocess Theory (McDaniel and Einstein, 2000), which was that there would be clusters of activation around cues but left open the possibility of different activations in the periods between spontaneous retrievals, linked to environmental *monitoring*. Prima facie the distinction between rostral and lateral PFC activations found between the conditions might be easiest to align with such a multiprocess view also. However, much further experimentation is required. Nevertheless, the ecological experimental design, wireless neuroimaging technique, brain-first analysis method, and novel PFC findings (i.e., spatial-temporal dynamics of functional activation in this naturalistic setting) do, we hope, represent a promising future direction towards testing complex cognitive models of higher cognition in naturalistic situations, thereby closing the distance between lab findings from purely experimental tasks and understanding brain processing in everyday life.

## Funding

This research was supported by grants from the 10.13039/100010269Wellcome Trust (104580/Z/14/Z), 10.13039/501100000781ERC (grant 313398-INTERACT) and the UK Medical Research Council (MRC MR/S003134/1). The content is solely the responsibility of the authors and does not necessarily represent the official views of the Wellcome Trust or other funding agencies.

## Data availability

All data reported in this paper are available upon request from the corresponding author.

## CRediT authorship contribution statement

**Paul W. Burgess:** Conceptualization, Methodology, Supervision, Writing – original draft. **James Crum:** Formal analysis, Data curation, Software, Writing – original draft, Writing – review & editing, Validation, Visualization. **Paola Pinti:** Investigation, Data curation, Formal analysis, Software, Visualization, Writing – review & editing. **Clarisse Aichelburg:** Investigation. **Dominic Oliver:** Investigation. **Frida Lind:** Investigation. **Sarah Power:** Investigation. **Elizabeth Swingler:** Investigation. **Uzair Hakim:** Writing – review & editing. **Arcangelo Merla:** Investigation. **Sam Gilbert:** Supervision. **Ilias Tachtsidis:** Funding acquisition, Supervision, Writing – review & editing. **Antonia Hamilton:** Supervision, Funding acquisition, Writing – review & editing.

## Declaration of Competing Interest

The authors declare no competing financial interests.

## Appendix Supplementary materials

Image, application 1
