# Supplementary material for: Prefrontal cortical activation associated with prospective memory while walking around a real-world street environment
Source: Neuroimage. 2022 Sep;258:119392. doi: 10.1016/j.neuroimage.2022.119392 (PMC10509823; doi:10.1016/j.neuroimage.2022.119392)
Supplement: Supplementary file 1 [file mmc1.docx]

**Supplementary Material**


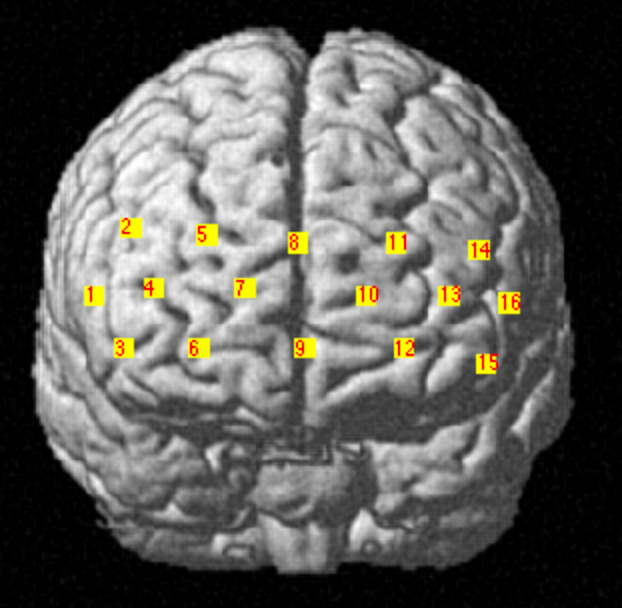


**Figure 9.** Group median channels location as determined by following the approach described in Zhang, X., Noah, J. A., Dravida, S., and Hirsch, J. (2017). Signal processing of functional NIRS data acquired during overt speaking. Neurophotonics 4(4), 041409. The approximate MNI co-ordinates of these positions are given in Table 3.

**Table 3.** *Median channel locations across all participants.*

| **Channel** | **X** | **Y** | **Z** | **BA-anatomy** | **Probability** |
| --- | --- | --- | --- | --- | --- |
| 1 | 54 | 38 | 12 | 45- pars triangularis Broca’s area | 0.96 |
| 2 | 45 | 46 | 30 | 45- pars triangularis Broca’s area | 0.53 |
|  |  |  |  | 46- dorsolateral prefrontal cortex | 0.47 |
| 3 | 48 | 54 | -2 | 46- dorsolateral prefrontal cortex | 0.91 |
| 4 | 39 | 61 | 13 | 10- frontopolar area | 0.61 |
|  |  |  |  | 46- dorsolateral prefrontal cortex | 0.39 |
| 5 | 26 | 62 | 27 | 10- frontopolar area | 0.53 |
|  |  |  |  | 46- dorsolateral prefrontal cortex | 0.38 |
| 6 | 29 | 69 | -2 | 10- frontopolar area | 0.26 |
|  |  |  |  | 11- orbitofrontal area | 0.74 |
| 7 | 16 | 72 | 13 | 10- frontopolar area | 1 |
| 8 | 2 | 65 | 26 | 10- frontopolar area | 0.98 |
| 9 | 1 | 72 | -2 | 10- frontopolar area | 0.84 |
| 10 | -16 | 72 | 11 | 10- frontopolar area | 1 |
| 11 | -25 | 62 | 27 | 10- frontopolar area | 0.47 |
|  |  |  |  | 46- dorsolateral prefrontal cortex | 0.48 |
| 12 | -27 | 68 | -3 | 10- frontopolar area | 0.30 |
|  |  |  |  | 11- orbitofrontal area | 0.70 |
| 13 | -38 | 61 | 12 | 10- frontopolar area | 0.54 |
|  |  |  |  | 46- dorsolateral prefrontal cortex | 0.46 |
| 14 | -45 | 47 | 25 | 45- pars triangularis Broca’s area | 0.59 |
|  |  |  |  | 46- dorsolateral prefrontal cortex | 0.41 |
| 15 | -47 | 51 | -5 | 46- dorsolateral prefrontal cortex | 0.89 |
| 16 | -53 | 39 | 9 | 45- pars triangularis Broca’s area | 0.94 |

*Note. The MNI coordinates (X, Y, Z) were used to identify the anatomical brain region covered by each channel using the Brodmann Area (BA) atlas. The probability that each channel covers the corresponding BA is listed in the last column (only probabilities >0.2 (i.e., 20%) are included).*
